# Data quality of reported child immunization coverage in 194 countries between 2000 and 2019

**DOI:** 10.1371/journal.pgph.0000140

**Published:** 2022-02-03

**Authors:** Cornelius Rau, Daniel Lüdecke, Laure B. Dumolard, Jan Grevendonk, Brenton M. Wiernik, Robin Kobbe, Marta Gacic-Dobo, M. Carolina Danovaro-Holliday

**Affiliations:** 1 Immunization Analysis & Insights (IAI), Department of Immunization, Vaccines and Biologicals (IVB), World Health Organization, Geneva, Switzerland; 2 Division of Neonatology and Pediatric Intensive Care, Department of Pediatrics, University Medical Center Hamburg-Eppendorf, Hamburg, Germany; 3 Institute of Medical Sociology, University Medical Center Hamburg-Eppendorf, Hamburg, Germany; 4 Department of Psychology, University of South Florida, Tampa, FL, United States of America; 5 Division of Infectious Diseases, First Department of Medicine, University Medical Center Hamburg-Eppendorf, Hamburg, Germany; University of Malaya Faculty of Medicine, MALAYSIA

## Abstract

Analyzing immunization coverage data is crucial to guide decision-making in national immunization programs and monitor global initiatives such as the Immunization Agenda 2030. We aimed to assess the quality of reported child immunization coverage data for 194 countries over 20 years. We analyzed child immunization coverage as reported to the World Health Organization (WHO) and the United Nations Children’s Fund (UNICEF) between 2000–2019 by all WHO Member States for Bacillus Calmette-Guérin (BCG) vaccine birth dose, first and third doses of diphtheria-tetanus-pertussis-containing vaccine (DTP1, DTP3), and first dose of measles-containing vaccine (MCV1). We assessed completeness, consistency, integrity, and congruence and assigned data quality flags in case anomalies were detected. Generalized linear mixed-effects models were used to estimate the probability of flags worldwide and for different country groups over time. The probability of data quality flags was 18.2% globally (95% confidence interval [CI] 14.8–22.3). The lowest probability was seen in South-East Asia (6.3%, 3.3–11.8, p = 0.002), the highest in the Americas (29.7%, 22.7–37.9, p < 0.001). The probability of data quality flags declined by 5.1% per year globally (3.2–7.0, p < 0.001). The steepest decline was seen in Africa (-9.6%, -13.0 to -5.8, p < 0.001), followed by Europe (-5.4%, -9.2 to -1.6, p = 0.0055), and the Americas (-4.9%, -9.2 to -0.6, p = 0.026). Most country groups showed a statistically significant decline, and none had a statistically significant increase. Over the past two decades, the quality of global immunization coverage data appears to have improved. However, progress has not been universal. The results highlight the need for joint efforts so that all countries collect, report, and use high-quality data for action in immunization.

## 1. Introduction

High-quality data are key in public health and development programs [1–3]. The global community has repeatedly highlighted the need for better data to track progress and ensure accountability of global initiatives such as the Global Strategy for Women’s, Children’s and Adolescents’ Health, Universal Health Coverage, and the Sustainable Development Goals [4–9]. The COVID-19 pandemic has illustrated that well-functioning health information systems and high-quality data are crucial to prevention, provision of care, and the successful rollout of new vaccines [10].

In immunization programs, data are needed to guide decision-making and monitor performance at the local, regional, and global level. Performance is often measured in terms of immunization coverage, that is the proportion of vaccinated individuals in the target population for a specific vaccine dose. Since the inception of the World Health Organization’s (WHO) Expanded Programme on Immunization (EPI) in 1974 and the adoption of the Global Vaccine Action Plan (GVAP) in 2012, estimated coverage rates have increased worldwide but plateaued around 85% between 2010 and 2019 [11–14]. Early estimates suggest major backslides during the COVID-19 pandemic [15, 16]. Technical immunization advisory groups have repeatedly expressed concern about immunization data quality since the turn of the millennium [11, 17–20]. Gavi, the Vaccine Alliance, cited data quality problems in 2020 as a “very high risk” to their investments [21].

In 2020, WHO Member States endorsed the *Immunization Agenda 2030 (IA2030)*: *a Global Strategy to Leave No One Behind* [22, 23]. Data-guidance is a core principle to achieve the vision of a “world where everyone, everywhere, at every age fully benefits from vaccines for good health and well-being” [24, 25]. The IA2030 highlights the need for improved data, and a strong base of analysis is essential to implement an effective Monitoring and Evaluation Framework for the Agenda [26]. However, the current extent of quality problems in reported immunization data remains unclear. A WHO Strategic Advisory Group of Experts (SAGE) Working Group on the Quality and Use of Global Immunization and Surveillance Data and two literature reviews have found a paucity of evidence on immunization data quality [27–30].

This study aimed to assess the quality of reported child immunization coverage data for 194 countries between 2000–2019.

## 2. Materials and methods

### 2.1 Data sources

All WHO Member States yearly report their childhood immunization coverage to WHO and the United Nations Children’s Fund (UNICEF) via the Joint Reporting Form on Immunization (JRF) [31]. Countries can report two types of data on every vaccine dose in the national immunization schedule: administrative coverage and official coverage [32]. Administrative coverage (or admin coverage) is calculated by dividing the number of children who received a specific vaccine dose during a reported year (numerator) by the number of children who should have received the vaccine (denominator). Countries usually obtain the numerator from aggregated doses administered in health facilities and use census projections or similar information to estimate the denominators. In contrast, official coverage comprises a percentage only and is a country’s best estimate of the coverage reached [28]. Thus, reporting official coverage is an opportunity for countries to adjust administrative data or report data from nationally representative surveys or another well-explained source. All countries are expected to report at least one type of data. Countries that do not have a centralized immunization reporting system often only report official estimates [28].

We gathered official and administrative coverage (percentage), number of doses given (numerator), and number of children in the target group (denominator) for Bacillus Calmette-Guérin (BCG) birth dose, first and third dose of diphtheria-tetanus-pertussis-containing vaccine (DTP1, DTP3), and first dose of measles-containing vaccine (MCV1) as reported to WHO and UNICEF by all WHO Member States for 20 years (2000–2019), as of 15 July 2020. For year-to-year comparisons, data for the year 1999 were also considered. The four vaccines were selected because they are almost universally used at birth (BCG), or in infancy as part of a basic vaccination series (MCV1, DTP1, DTP3).

### 2.2 Country classifications

Several grouping classifications were used to examine differences in the data quality between different groups of countries, based on demographic, economic, and political indicators (see S1 Appendix). Estimates of the countries’ total population, live births cohort, and surviving infants cohort were obtained from the United Nations Population Division (UNPD) [33]. Information on WHO World Regions, immunization schedules and WHO/UNICEF Estimates on National Immunization Coverage (WUENIC) were provided by WHO [34, 35]. Estimates of infant mortality rate (IMR), that is the probability of dying between birth and one year of age per 1000 live births, were obtained from the UN Inter-agency Group for Child Mortality Estimation (IGME), as of September 2020 [36]. Historical classification of countries by income based on gross national income per capita in 2000–2019 [37], and fragile and conflict-affected situations (FCS) in 2004–2019 [38] were derived from the World Bank, as of July 2020. Latest estimates of birth registration levels, that is the percentage of children under age five whose births are registered, were obtained from UNICEF, as of July 2020 [39]. In addition, we classified countries by whether they had received financial support from Gavi, the Vaccine Alliance, between 2001–2019 [40].

### 2.3 Definition of data quality

There is no universally accepted definition of data quality in immunization programs [28, 29]. For this analysis, we adopted an approach by Bloland and MacNeil [41] and the SAGE Working Group on Immunization Data Quality [28] that defined high-quality data as “accurate, precise, relevant, complete and timely enough for the intended purpose” (Table 1). We used this working definition, acknowledging that the dimensions of timeliness and relevancy were not verifiable directly using the available data.

**Table 1:** Dimensions of data quality in immunization programs.

Dimension	Definition
**Accuracy**	Closeness of a measurement or estimate to the true value
• Congruence (proxy)	Degree to which data obtained from different sources agree with each other
• Integrity (proxy)	Degree to which data are unchanged from the original
**Precision**	Degree of spread of a series of measurements that is independent of accuracy
• Consistency (proxy)	Degree to which data attributes are free from contradiction, large fluctuations over time, and coherent with other data in a specific context of use
**Relevancy**	Degree to which data reflect what is most important for decision-making
**Completeness**	Whether or not all relevant data needed for decision-making are available for use
**Timeliness**	Degree to which data are current and available when needed to inform decisions

Notes: Adapted from Bloland and MacNeil and the SAGE Working Group on the Quality and Use of Immunization and Surveillance Data [28, 41].

### 2.4 Data quality checks

We assessed the quality of reported immunization coverage data in terms of completeness, consistency, integrity, and congruence, based on existing recommendations by WHO and the SAGE Working Group combined with our methodology as defined below [28, 42–46]. We used a stepwise approach that included 1) checking country data for any given anomaly, 2) flagging summaries of data that contained anomalies, and 3) modeling the probability of flags for groups of countries.

First, a series of data quality checks were applied to the reported data, with each check related to one of the four dimensions of data quality assessed. Forty-two countries without BCG birth dose in the national routine immunization schedule were excluded from analyses involving BCG vaccine. Eleven countries with a total population of fewer than 90,000 people in 2019 were excluded from checks comparing denominators with external sources because UNPD does not provide estimates of births and surviving infants for these countries. Fourteen countries that do not use a centralized immunization reporting system were excluded from analyses involving admin coverage, numerators, or denominators. Finally, we only considered data for years with membership for countries that became WHO Member States during the study period. Thus, data from Timor-Leste were assessed beginning in 2002, from Montenegro beginning in 2006, and from South Sudan beginning in 2011.

#### 2.4.1 Completeness

Completeness was assessed by calculating proportions of missing numerators, denominators, admin and official percent coverage.

#### 2.4.2 Congruence

Congruence was assessed by comparing BCG denominators to UNPD estimates of live births with deviations of ≥10% considered abnormal. The implied infant mortality rate (IIMR) was calculated as

$$Implied\;infant\;mortality\;rate=\frac{number\;of\;live\;births-number\;of\;surviving\;infants}{number\;of\;live\;births}$$

using BCG denominators as a proxy for the number of live births and DTP1, DTP3, and MCV1 denominators, respectively, as proxies for the number of surviving infants [42]. Implied infant mortality rates that were zero or negative, or outside the 90% uncertainty intervals (UI) of UN-IGME estimates, and denominators for DTP1, DTP3, or MCV1 differing by ≥10% from UNPD estimates of surviving infants were considered abnormal.

#### 2.4.3 Consistency

Consistency was assessed by identifying same numerators or denominators as reported in the preceding year to detect potential copying and pasting of previous year data, coverage levels equal or over 100%, and data differing from year to year by ≥10%. Dropout rates for numerators and coverage levels between DTP1 and DTP3 were calculated as

$$DTP\;dropout\;rate\;(\%)=\frac{DTP1-DTP3\;}{DTP1\;}\times 100$$

and considered abnormal if zero or negative.

#### 2.4.4 Integrity

Integrity was assessed by recalculating admin coverage as

$$Recalculated\;administrative\;coverage\;(\%)=\frac{numerator}{denominator}\times 100.$$

If the recalculated admin coverage was ≥100% or did not match the admin coverage, the numerator and denominator were considered abnormal.

### 2.5 Flagging coverage reports

**Fig 1:**
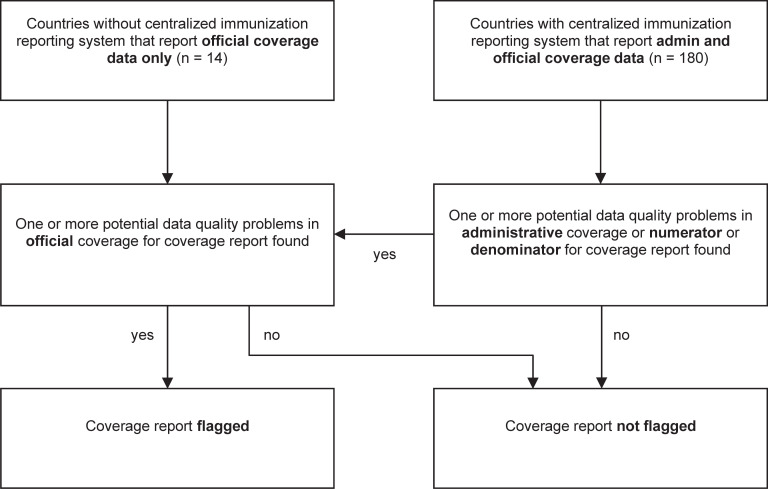
Method for flagging the quality of reported immunization data by type of country immunization reporting system for 194 WHO Member States. Notes: Countries without a centralized immunization reporting system were: Andorra, Austria, Belgium, Canada, Finland, France, Germany, Greece, Luxemburg, Monaco, Norway, Sweden, Switzerland, and the United States of America. For detailed flowcharts listing all data quality checks for each vaccine dose by type of country immunization reporting system see S1-S4 Figs in S1 Appendix.

To account for the heterogeneous landscape of immunization systems among countries, we summarized country-provided information on each vaccine dose for every year as one country-year-vaccine coverage report (hereinafter referred to as “coverage report”). A coverage report was flagged if at least one data quality check in the underlying data revealed anomalies. To prevent countries that reported both admin and official data on the same vaccine dose and year from being disadvantaged in case of anomalies found in admin but not in official data, official data that passed the data quality checks could outweigh anomalies in admin data (Fig 1 and S1-S4 Figs in S1 Appendix).

### 2.6 Statistical analysis

We modeled the probability of data quality flags worldwide, for different vaccine doses, and separate country groups over time using generalized linear mixed-effects models. The modeling strategy is described in detail in the online supplement (see S1 Appendix).

The country variable was used as level-two random effect in all models to account for variation between countries. Year and vaccine dose were used as random slopes to allow for varying effects between countries.

First, we estimated the global probability of data quality flags pooled for all years using an intercept-only model including random effects only. Then, using a main model, we calculated the probability of data quality flags by vaccine dose, WHO World Region, World Bank income group, quintile of total population size, immunization coverage level, and support by Gavi, the Vaccine Alliance. Due to limited data availability, two additional models were calculated that included the same variables as previously mentioned plus the variable birth registration level and fragile- and conflict-affected situations (FCS) status, respectively.

Second, we estimated the global time trend using a model without interaction terms. Again, we used a main model to calculate the probability of data quality flags for each vaccine dose and country group plus additional models for birth registration level and FCS status.

Third, the same models were fitted; however, the predictor for year used in the interaction was modeled as a spline with five degrees of freedom to allow plotting over time.

All analyses were conducted using the R language for statistical computing, version 4.0.2 [47]. The glmmTMB package version 1.0.2.1 was used to fit mixed-effects models [48]. Predicted time trends for data quality flags were calculated using the emmeans package [49] version 1.5.2.1 and ggeffects package [50] version 0.16.0. The a priori significance level was set at p = 0.05.

## 3. Results

### 3.1 Data quality checks

All 194 Member States reported immunization coverage data to WHO and UNICEF between 2000–2019. However, our data quality checks revealed anomalies in 47% (26390/55836) of expected data points involving all reporting countries but one. See Table 2 for descriptive results of data quality checks.

**Table 2:** Immunization coverage data points and reporting countries affected by potential data quality issues, by dimension of data quality, 194 WHO Member States, 2000–2019.

	Data points affected/data points reported (%)		Countries affected/countries reporting (%)	
**Completeness**				
Type of data point missing				
Admin coverage missing	1123/13744	(8%)	124/180	(69%)
Denominator missing	1445/13744	(11%)	134/180	(74%)
Official coverage missing	1609/14604	(11%)	150/194	(77%)
Numerator missing	1532/13744	(11%)	137/180	(76%)
Total data points missing	5709/55836	(10%)	172/194	(89%)
**Congruence **				
DTP3 denominator versus UNPD surviving infants ≥10%	1042/3104	(34%)	139/171	(81%)
MCV1 denominator versus UNPD surviving infants ≥10%	1046/3049	(34%)	145/171	(85%)
DTP1 denominator versus UNPD surviving infants ≥10%	973/2826	(34%)	132/166	(80%)
BCG denominator versus UNPD live births ≥10%	1061/2846	(37%)	133/161	(83%)
Implied IMR using MCV1 zero or negative	1176/2892	(41%)	159/168	(95%)
Implied IMR using DTP1 zero or negative	1304/2761	(47%)	159/166	(96%)
Implied IMR using DTP3 zero or negative	1416/2940	(48%)	162/169	(96%)
Implied IMR using DTP1 outside 90% UI of UN-IGME IMR	2315/2634	(88%)	158/158	(100%)
Implied IMR using DTP3 outside 90% UI of UN-IGME IMR	2483/2813	(88%)	161/161	(100%)
Implied IMR using MCV1 outside 90% UI of UN-IGME IMR	2446/2769	(88%)	160/160	(100%)
**Consistency **				
Same numerator as in preceding year	58/11389	(1%)	22/179	(12%)
Same denominator as in preceding year	269/11468	(2%)	51/180	(28%)
Official coverage ≥100%	588/12995	(5%)	88/194	(45%)
Admin coverage ≥100%	998/12621	(8%)	104/180	(58%)
Recalculated admin coverage ≥100%	1125/12159	(9%)	118/180	(66%)
Denominator year-to-year difference ≥10%	1404/11360	(12%)	150/180	(83%)
Official coverage year-to-year difference ≥10%	1579/12191	(13%)	133/194	(69%)
Admin coverage year-to-year difference ≥10%	1651/12018	(14%)	139/178	(78%)
DTP1 to DTP3 doses dropout rate zero or negative	486/2945	(17%)	113/173	(65%)
DTP1 to DTP3 admin coverage dropout rate zero or negative	623/3027	(21%)	125/174	(72%)
DTP1 to DTP3 official coverage dropout rate zero or negative	662/3085	(21%)	136/187	(73%)
**Integrity**				
Admin coverage different from recalculated admin coverage	1214/12116	(10%)	169/180	(94%)
**All checks combined**				
Type of data point				
Numerator	3569/13744	(26%)	176/180	(98%)
Official coverage	4814/14604	(33%)	192/194	(99%)
Administrative coverage	5439/13744	(40%)	180/180	(100%)
Denominator	12568/13744	(91%)	180/180	(100%)
Total	26390/55836	(47%)	193/194	(99%)

Notes: Country data as reported by 15 July 2020. The number of combined data quality checks applied to each type of data point was not equal, thus data should be interpreted with caution. BCG = Bacillus Calmette-Guérin vaccine birth dose. DTP1 = first dose of diphtheria-tetanus-pertussis-containing vaccine. DTP3 = third dose of diphtheria-tetanus-pertussis-containing vaccine. IMR = infant mortality rate. MCV1 = first dose of measles-containing vaccine. UI = uncertainty interval. UN-IGME = United Nations Inter-agency Group for Child Mortality Estimation. UNPD = United Nations Population Division.

Completeness rates were similar among different types of data. Where data were available, checks for congruence showed the highest frequencies of anomalies, followed by consistency and integrity.

Denominators were most affected with a 91% (12568/13744) rate of abnormal data quality check results, followed by admin coverage with 40% (5439/13744), official coverage with 33% (4814/14604), and numerators with 26% (3569/13744).

Over the period analyzed, DTP1 was the vaccine dose most affected by data quality issues with a 59% (8646/14604) rate of abnormal data quality check results, followed by DTP3 with 48% (6977/14604), BCG with 41% (4965/12024), and MCV1 with 40% (5802/14604). See S1 Table in S1 Appendix.

### 3.2 Modeled probability of data quality flags

**Fig 2:**
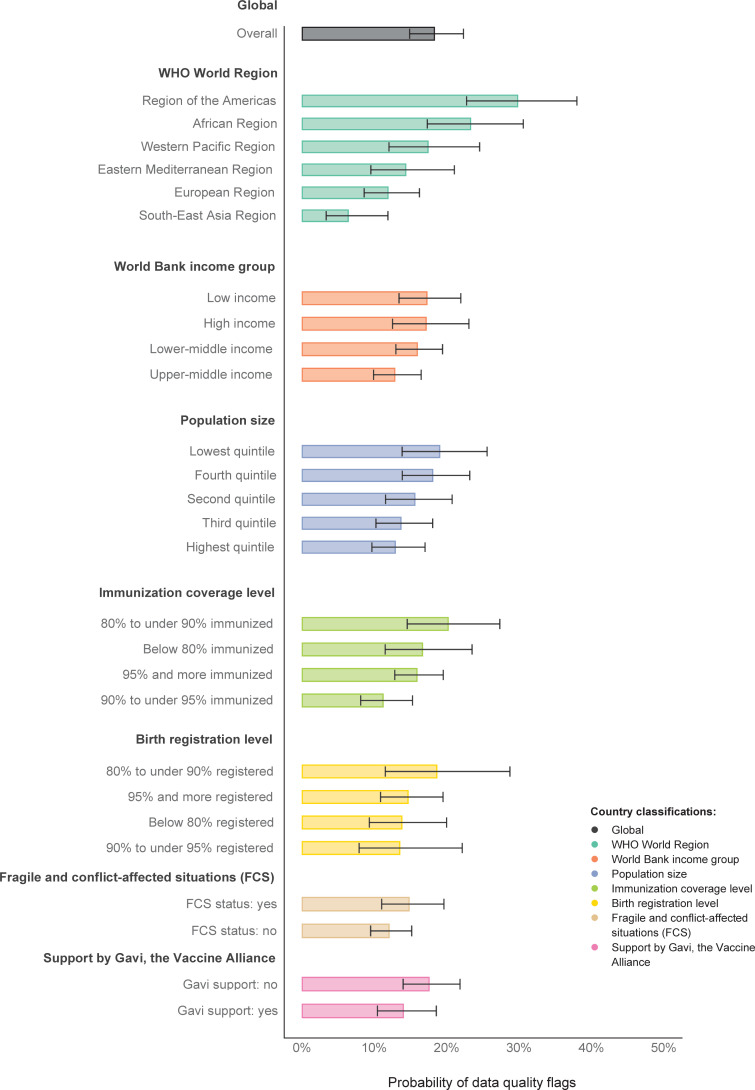
Modeled probability of data quality flags for immunization coverage reports for DTP1, DTP3, MCV1, and BCG worldwide and by different country classifications, 194 WHO Member States, 2000–2019. Notes: Error bars represent 95% confidence intervals (CI). Country data as reported by 15 July 2020. BCG = Bacillus Calmette-Guérin vaccine birth dose. DTP1 = first dose of diphtheria-tetanus-pertussis-containing vaccine. DTP3 = third dose of diphtheria-tetanus-pertussis-containing vaccine. FCS = fragile and conflict-affected situations. MCV1 = first dose of measles-containing vaccine. Countries were grouped separately for each year by World Bank income groups, population size, and fragile and conflict-affected situations (FCS) classification. All other groupings were done for all years together. FCS status was available for 2004–2019 only. Immunization coverage level was based on average DTP1 and DTP3 coverage estimated by WHO and UNICEF for 2017–2019, as of July 2020. Birth registration levels refer to children under age five who have been registered based on the latest available UNICEF estimate. Support by Gavi, the Vaccine Alliance, refers to funding in any year between 2000–2019. See S1 Appendix for detailed lists of countries.

The overall probability of data quality flags in immunization coverage reports was 18.2% globally (95% confidence interval [CI] 14.8–22.3) between 2000–2019 (Fig 2 and S2 Table in S1 Appendix).

Three out of six WHO Regions showed a statistically significant difference from the mean of this country classification. South-East Asia had a significantly lower probability (6.3%, 3.3–11.8, p = 0.002). The highest probabilities were seen in the Americas (29.7%, 22.7–37.9, p < 0.001) and in Africa (23.2%, 17.3–3.5, p = 0.013). Upper-middle income countries had a statistically significant lower probability of data quality flags than the classification mean (12.8%, 9.8–16.4, p = 0.033). Countries with an immunization coverage level of 90% to under 95% had a statistically significant lower probability of data quality flags than the classification mean (11.2%, 8.1–15.2, p = 0.024).

There was no statistically significant difference in the probability of data quality flags by population size quintile, birth registration level, FCS status, or support by Gavi, the Vaccine Alliance, and the corresponding classification mean.

The probability of data quality flags across vaccine doses ranged from 8.2% for BCG birth dose (95% CI 6.1–10.8, p < 0.001) to 36.0% for DTP1 (95% CI 29.8–42.6, p < 0.001). Coverage reports for BCG birth dose and for MCV1 (9.0%, 95% CI 7.1–11.5, p < 0.001) had a statistically significant lower probability of data quality flags, while reports for DTP1 and DTP3 (19.3%, 95% CI 15.9–23.3, p < 0.001) had a statistically significant higher probability of data quality flags (S3 Table and S5 Fig in S1 Appendix).

### 3.3 Trends of the probability of data quality flags

**Fig 3:**
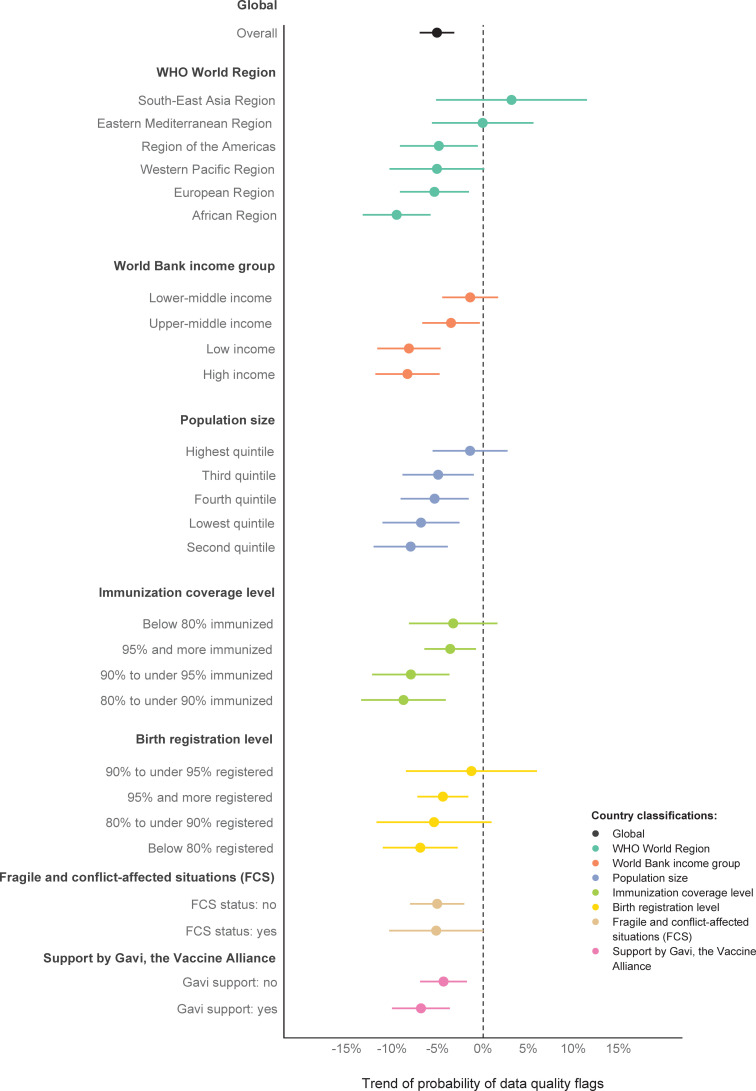
Modeled trends of the probability of data quality flags for immunization coverage reports for DTP1, DTP3, MCV1, and BCG, by different country classifications, 194 WHO Member States, 2000–2019. Notes: Colored lines represent 95% confidence intervals (CI). Country data as reported by 15 July 2020. BCG = Bacillus Calmette-Guérin vaccine birth dose. DTP1 = first dose of diphtheria-tetanus-pertussis-containing vaccine. DTP3 = third dose of diphtheria-tetanus-pertussis-containing vaccine. FCS = fragile and conflict-affected situations. MCV1 = first dose of measles-containing vaccine. Countries were grouped separately for each year by World Bank income groups, population size, and fragile and conflict-affected situations (FCS) classification. All other groupings were done for all years together. FCS status was available for 2004–2019 only. Immunization coverage level was based on average DTP1 and DTP3 coverage estimated by WHO and UNICEF for 2017–2019, as of July 2020. Birth registration levels refer to children under age five who have been registered based on the latest available UNICEF estimate. Support by Gavi, the Vaccine Alliance, refers to funding in any year between 2000–2019. See S1 Appendix for detailed lists of countries.

**Fig 4:**
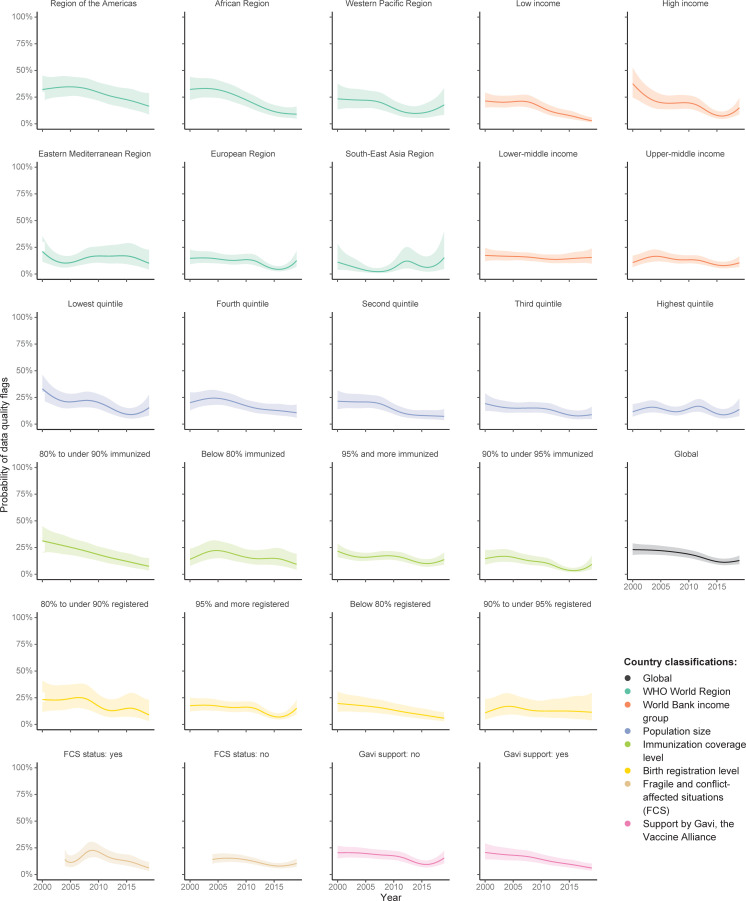
Modeled trends of the probability of data quality flags for immunization coverage reports for DTP1, DTP3, MCV1, and BCG, by different country classifications, 194 WHO Member States, 2000–2019. Notes: Shading represents 95% confidence intervals (CI). Country data as reported by 15 July 2020. BCG = Bacillus Calmette-Guérin vaccine birth dose. DTP1 = first dose of diphtheria-tetanus-pertussis-containing vaccine. DTP3 = third dose of diphtheria-tetanus-pertussis-containing vaccine. FCS = fragile and conflict-affected situations. MCV1 = first dose of measles-containing vaccine. Countries were grouped separately for each year by World Bank income groups, population size, and fragile and conflict-affected situations (FCS) classification. All other groupings were done for all years together. FCS status was available for 2004–2019 only. Immunization coverage level was based on average DTP1 and DTP3 coverage estimated by WHO and UNICEF for 2017–2019, as of July 2020. Birth registration levels refer to children under age five who have been registered based on the latest available UNICEF estimate. Support by Gavi, the Vaccine Alliance, refers to funding in any year between 2000–2019. See S1 Appendix for detailed lists of countries.

The global probability of data quality flags declined by 5.1% per year (95% CI 3.2–7.0, p < 0.001) between 2000–2019 (Figs 3 and 4, and S4 Table in S1 Appendix).

Three out of six WHO World Regions showed a statistically significant decrease in the probability of data quality flags. The steepest decline was seen in Africa (-9.6%, -13.0 to -5.8, p < 0.001), followed by Europe (-5.4%, -9.2 to -1.6, p = 0.0055), and the Americas (-4.9%, -9.2 to -0.6, p = 0.026).

All other country groups showed statistically significant declines except lower-middle income countries, countries within the highest quintile of population size, countries with an immunization coverage level below 80%, countries with a birth registration rate between 80% to under 95%, and fragile and conflict-affect situations (FCS). No country group showed a statistically significant overall increase. However, some groups experienced a rise in the year 2019 (Fig 4).

The yearly trend of the probability of data quality flags across vaccine doses ranged from -6.7% for DTP1 (95% CI -9.1 to -4.4, p < 0.001) to -4.3% for MCV1 (95% CI -6.9 to -1.7, p < 0.001). All vaccine doses saw a statistically significant decline (S5 Table and S6 Fig in S1 Appendix).

## 4. Discussion

This study provides a comprehensive quality assessment of child immunization coverage data for all 194 WHO Member States using publicly available reported data.

About one in five vaccine coverage reports sent to WHO/UNICEF between 2000–2019 contained data that warrant further quality investigation. Across WHO World Regions, South-East Asia had the lowest rate of potential data quality issues, while the share of quality flags in reports from the Americas and Africa was higher than the global mean. In addition, we found fewer potential data quality issues in data from countries with an immunization coverage level of 90% to under 95%, and upper-middle income countries. Reports of BCG and MCV1 had lower rates of potential data quality problems, whereas DTP1 and DTP3 had higher rates.

The overall findings were consistent with previous evidence [18, 51–60]. However, most existing studies were limited to specific groups of countries [52–55, 59], or to comparisons with external data such as household surveys [52, 54, 55], or with population estimates [56, 58–60]. Compared to prior work, this analysis found lower rates of consistency problems. For example, previous assessments by WHO and Stashko et al. found year-to-year differences of at least ten percent in up to 20% of coverage data, compared to 14% for admin coverage and 13% for official coverage in this study [51, 60]. A 2009 assessment in the Americas found negative dropout rates between DTP1 and DTP3 doses in 34% of cases [18], compared to 17% in this study. In contrast, the frequency of congruence and completeness issues was similar to previous analyses. For example, the proportion of deviations of BCG denominators from UNPD live birth estimates in the literature ranged from 27% in the Americas [18], to 37% globally [60], and 49% in Sub-Saharan Africa [61], with the finding of 37% in this study in between. Stashko et al. saw admin coverage levels of 100% or above in 6%–11% of reporting events, compared to 8% in this assessment [60].

While existing evidence repeatedly found data quality issues in immunization coverage data, the underlying causes have rarely been investigated [27, 29]. For example, population groups may be systematically excluded from accessing care, or data from people vaccinated in the private sector may never be reported and thus not included in the calculation of administrative coverage [29]. Some studies have used household surveys to assess reported coverage. Although surveys are helpful to complement program data, they cannot guide program management alone as they are infrequent, expensive, often limited in geographic representation, and themselves not free from data quality concerns [62]. Official coverage levels reported to WHO and UNICEF may not contain any accompanying explanation, and what approach of estimation is used or how the latter changes over time may be unknown [28]. Coverage may drop or increase by more than 10% from one year to the next if a vaccine stock-out or a decline in vaccine acceptance occurs [30]. The same can occur to target population estimates due to mass migration, conflict, or humanitarian crises [28, 42, 60]. Data can also fluctuate when dealing with small population sizes [42]. Thus, interpreting quality in countries with small populations needs to be done with caution.

This study found potential data quality issues in all country groups. However, further studies are needed to explore the underlying causes. Further exploration is required to investigate likely differences in the data quality across vaccines, especially whether the wide use of DTP coverage as a marker of immunization performance plays a role [28, 63]. In-depth analyses of the relationship between data quality and potential inequality factors, such as subnational state or district, ethnicity, and gender, are also needed. Some country groups showed an apparent upward trend in potential data quality issues in 2019. WHO and UNICEF collected the data for 2019 in the second quarter of 2020. Research is needed to determine whether increased rates of potential data quality problems in 2019 may be due to limited capacity for reporting during the COVID-19 pandemic in 2020. It should also be explored whether 2020 data collected in 2021 was more affected by delays and inconsistencies.

While our analysis examined data quality from a global perspective, identifying problematic data is a challenge once it has entered the reporting chain. Consequently, data quality assessments should be conducted at all levels, beginning at the point of vaccination. Here, data are often not available, not analyzed, or not suitable for decision-making [1, 64]. Time-consuming or duplicative documentation can overburden health workers and inhibit efficient workflows [2]. In addition, data quality might suffer if people are not empowered to use and benefit from the data they collect. Innovative tools, better guidance, and continuous on-the-job training are needed to facilitate change and create a “data use culture” and promote "data literacy" [44]. Further, operational research should be promoted to identify barriers to change and create evidence-based interventions to increase data quality [65, 66]. This should include learning from other health sectors and programs. For example, research on target population estimates for antenatal care or mass drug administration such as vitamin A supplementation or deworming campaigns can guide quality improvements of denominators in immunization campaigns [44].

While the quality of global immunization coverage data has improved, countries have not made equal progress. More could be learned from WHO World Regions and countries that have reduced quality flags. Moving forward, the global community should prioritize lower-middle income countries, larger countries, fragile and conflict-affected situations, and countries with low immunization performance to improve immunization data quality.

Tackling data quality requires increased commitment from stakeholders at all levels, including national governments, the non-profit sector, and organizations within the UN system [3]. Building on previous guidance [67–71], WHO has published a technical package of five essential interventions in 2021 to strengthen country health information systems, including advice on tools and standards to improve data quality [2, 72]. Also, WHO and UNICEF have launched a cloud-based platform for countries to check and report their immunization data online (eJRF) which replaced the Excel-based Joint Reporting Form on Immunization [73] featuring pre-populated historical data, real-time validation checks, automatic calculations, and a module to collect data on COVID-19 immunization rates monthly [73].

### 4.1 Strengths and limitations

This study has several strengths. First, to our knowledge, it is the first comprehensive global assessment of reported child immunization data quality since 1998 for all 194 WHO Member States. Second, it uses a systematic approach based on a multi-dimensional definition of quality and a set of checks derived from public recommendations. Third, we present global estimates on immunization coverage data quality, allowing for trend analysis and comparisons between country groups.

This study also has limitations. First, the lack of a gold standard for measuring immunization coverage makes it impossible to directly ascertain the data quality dimension of accuracy. Therefore, like other studies, we used congruence and integrity as proxies for measuring accuracy, and consistency as a proxy for measuring precision. Second, as the underlying cause for anomalies in the data was unknown, “false positive” flags may have been assigned, and some coverage reports that were not flagged may be problematic. Third, our analysis focused only on reported national-level coverage and the modeling strategy was designed to derive estimates for groups of countries, not for individual countries. Fourth, as the number of external data sources and quality checks available was not equal for all vaccine doses and types of data, comparisons between different vaccine doses and data types should be interpreted with caution. Finally, this analysis refers to a pre-COVID-19 immunization era. Data on the year 2020 may reveal further quality disturbances not yet captured by this study. Increasing trends in data quality problems in 2019 should be discussed cautiously, as these estimates are less well supported by surrounding values.

## 5. Conclusions

Over the past two decades, the quality of global immunization coverage data has improved. However, progress has not been universal. While Africa showed the fastest improvements, followed by Europe and the Americas, other country groups had stagnating proportions of potentially problematic data. These findings can inform national and international policy and action to strengthen national immunization information systems. The adoption of the Immunization Agenda 2030 provides momentum to enter the next era of global vaccination programs driven by high-quality data.

## Supporting information

S1 Appendix Online supplement including modeling strategy, supplementary tables, supplementary figures, and lists of countries. (DOCX)
