# Supplementary material for: Data quality of reported child immunization coverage in 194 countries between 2000 and 2019
Source: PLOS Glob Public Health. 2022 Feb 3;2(2):e0000140. doi: 10.1371/journal.pgph.0000140 (PMC10022119; doi:10.1371/journal.pgph.0000140)
Supplement: S1 Appendix — (DOCX) [file pgph.0000140.s001.docx]

**Appendix**

Data quality of reported child immunization coverage in 194 countries between 2000 and 2019

**Contents**

[1 Modeling strategy 2](#_Toc80570058)

[1.1 Probability of data quality flags 2](#_Toc80570059)

[1.2 Trends of the probability of data quality flags 3](#_Toc80570060)

[1.3 Plotting trends of the probability of data quality flags 3](#_Toc80570061)

[1.4 Software used 4](#_Toc80570062)

[2 Supplementary Tables 5](#_Toc80570063)

[3 Supplementary Figures 10](#_Toc80570064)

[4 Lists of countries 16](#_Toc80570065)

[4.1 WHO Member States 16](#_Toc80570066)

[4.2 WHO World Region 16](#_Toc80570067)

[4.3 World Bank income group 17](#_Toc80570068)

[4.4 Population size 19](#_Toc80570069)

[4.5 Immunization coverage level 21](#_Toc80570070)

[4.6 Birth registration level 22](#_Toc80570071)

[4.7 Fragile- and conflict-affected situations (FCS) 23](#_Toc80570072)

[4.8 Support by Gavi, the Vaccine Alliance 23](#_Toc80570073)

[4.9 Immunization reporting system 24](#_Toc80570074)

[4.10 Immunization schedule 25](#_Toc80570075)

[5 References 26](#_Toc80570076)

# Modeling strategy

To estimate the probability of data quality flags, we used generalized linear mixed-effects models with logit-link. The country-variable “iso” was used as level-two random effect in all models. We expected the probabilities of data quality flags to vary between countries over time, thus the variable “year” was used as random slope. Furthermore, we assumed that probabilities of data quality flags for the different vaccine doses could vary by country. Hence, “vaccine” was also used as random slope. However, we modeled “vaccine” as uncorrelated with the random intercept.

We considered the following country classifications: WHO World Region (“region”), World Bank income group (“income”), quintile of population size (“population.quintile”), immunization coverage level (“immunization.coverage”), birth registration level (“birth.registration”), fragile and conflict-affected situations (FCS-status, “fragile.state”), and Support by Gavi, the Vaccine Alliance (“gavi”).

## Probability of data quality flags

To estimate the probability of data quality flags globally (Figure 2: Global, Supplementary Table 2: Global), we calculated an intercept-only model:

|  | $outcome\sim\mathrm{Binomial}\left( 1, p_{ij} \right)$ | (1) |
| --- | --- | --- |

|  | $\mathrm{logit}\left( p_{ij} \right)=\beta_{0j}+\beta_{1j}\times year_{ij}+\beta_{2j}\times vaccine_{ij}$ | (2) |
| --- | --- | --- |

|  | $\left[ \begin{matrix} \beta_{0j} & \beta_{1j} & \beta_{2j} \end{matrix} \right]^{'}\sim\mathrm{MVN}\left( \left[ \begin{matrix} \gamma_{00} \\ \gamma_{10} \\ \gamma_{20} \end{matrix} \right],\left[ \begin{matrix} \tau_{0}^{2} & \phi_{01}\tau_{0}\tau_{1} & 0 \\ \phi_{01}\tau_{0}\tau_{1} & \tau_{1}^{2} & 0 \\ 0 & 0 & \tau_{2}^{2} \end{matrix} \right] \right)$ | (3) |
| --- | --- | --- |

In a second step, we calculated a main regression model to estimate the probability of data quality flags for each vaccine dose, and for each country group within each country classification (Figure 2, Supplementary Tables 2 and 3). We excluded the variable “birth.registration” from the main model due to the limited number of countries with available data (172/194 countries). Likewise, we excluded the variable “fragile.state” due to the limited number of years with available data (2004–2019). Instead, two additional models were calculated, including the main model plus the variables "birth.registration" and "fragile.state", respectively. To avoid replication, we only show the formula for the main model:

|  | $outcome\sim\mathrm{Binomial}\left( 1, p_{ij} \right)$ | (4) |
| --- | --- | --- |

|  | $\mathrm{logit}\left( p_{ij} \right)=\beta_{0j}+\beta_{1j}\times year_{ij}+\beta_{2j}\times vaccine_{ij}+\beta_{3}\times income_{i}+\beta_{4}\times region_{i}+\beta_{5}\times population. quintile_{i}+\beta_{6}\times gavi_{i}+\beta_{7}\times{immunization.coverage}_{i}$ | (5) |
| --- | --- | --- |

|  | $\left[ \begin{matrix} \beta_{0j} & \beta_{1j} & \beta_{2j} \end{matrix} \right]^{'}\sim\mathrm{MVN}\left( \left[ \begin{matrix} \gamma_{00} \\ \gamma_{10} \\ \gamma_{20} \end{matrix} \right],\left[ \begin{matrix} \tau_{0}^{2} & \phi_{01}\tau_{0}\tau_{1} & 0 \\ \phi_{01}\tau_{0}\tau_{1} & \tau_{1}^{2} & 0 \\ 0 & 0 & \tau_{2}^{2} \end{matrix} \right] \right)$ | (6) |
| --- | --- | --- |

## Trends of the probability of data quality flags

To estimate the yearly trend of the global probability of data quality flags (Figure 3: Global, Supplementary Table 4: Global), we calculated a model with only “year” as predictor:

|  | $outcome\sim\mathrm{Binomial}\left( 1, p_{ij} \right)$ | (7) |
| --- | --- | --- |

|  | $\mathrm{logit}\left( p_{ij} \right)=\beta_{0j}+\beta_{1ij}\times year_{ij}+\beta_{2j}\times vaccine_{ij}$ | (8) |
| --- | --- | --- |

|  | $\left[ \begin{matrix} \beta_{0j} & \beta_{1j} & \beta_{2j} \end{matrix} \right]^{'}\sim\mathrm{MVN}\left( \left[ \begin{matrix} \gamma_{00} \\ \gamma_{10} \\ \gamma_{20} \end{matrix} \right],\left[ \begin{matrix} \tau_{0}^{2} & \phi_{01}\tau_{0}\tau_{1} & 0 \\ \phi_{01}\tau_{0}\tau_{1} & \tau_{1}^{2} & 0 \\ 0 & 0 & \tau_{2}^{2} \end{matrix} \right] \right)$ | (9) |
| --- | --- | --- |

Again, we calculated a main regression model to estimate the yearly trends of the probability of data quality flags for each vaccine dose, and for each country group within each country classification (Figure 3, Supplementary Tables 4 and 5). We excluded the variable “birth.registration” from the main model due to the limited number of countries with available data (172/194 countries). Likewise, we excluded the variable “fragile.state” due to the limited number of years with available data (2004–2019). Instead, two additional models were calculated, including the main model plus the variables "birth.registration" and "fragile.state", respectively. To avoid replication, we only show the formula for the main model:

|  | $outcome\sim\mathrm{Binomial}\left( 1, p_{ij} \right)$ | (10) |
| --- | --- | --- |

|  | $\mathrm{logit}\left( p_{ij} \right)=\beta_{0j}+\beta_{1j}\times year_{ij}+\beta_{2}\times vaccine_{ij}+\beta_{3j}\times year_{ij}\times vaccine_{ij}+$ $=\beta_{4}\times income_{i}+\beta_{5}\times region_{i}+\beta_{6}\times population. quintile_{i}+\beta_{7}\times gavi_{i}+\beta_{8}\times i{mmunization.coverage}_{i}$ | (11) |
| --- | --- | --- |

|  | $\left[ \begin{matrix} \beta_{0j} & \beta_{1j} & \beta_{2j} \end{matrix} \right]^{'}\sim\mathrm{MVN}\left( \left[ \begin{matrix} \gamma_{00} \\ \gamma_{10} \\ \gamma_{20} \end{matrix} \right],\left[ \begin{matrix} \tau_{0}^{2} & \phi_{01}\tau_{0}\tau_{1} & 0 \\ \phi_{01}\tau_{0}\tau_{1} & \tau_{1}^{2} & 0 \\ 0 & 0 & \tau_{2}^{2} \end{matrix} \right] \right)$ | (12) |
| --- | --- | --- |

## Plotting trends of the probability of data quality flags

To detect varying patterns of probabilities of data quality flags over time, we fitted the same model as for the time trend with only “year” as predictor. However, the predictor for “year” used in the interaction was modeled as spline with five degrees of freedom (Figure 4: Global):

|  | $outcome\sim\mathrm{Binomial}\left( 1, p_{ij} \right)$ | (13) |
| --- | --- | --- |

|  | $\mathrm{logit}\left( p_{ij} \right)=\beta_{0j}+\beta_{1ij}\times year_{ij}+\beta_{2j}\times vaccine_{ij}$ | (14) |
| --- | --- | --- |

|  | $\left[ \begin{matrix} \beta_{0j} & \beta_{1j} & \beta_{2j} \end{matrix} \right]^{'}\sim\mathrm{MVN}\left( \left[ \begin{matrix} \gamma_{00} \\ \gamma_{10} \\ \gamma_{20} \end{matrix} \right],\left[ \begin{matrix} \tau_{0}^{2} & \phi_{01}\tau_{0}\tau_{1} & 0 \\ \phi_{01}\tau_{0}\tau_{1} & \tau_{1}^{2} & 0 \\ 0 & 0 & \tau_{2}^{2} \end{matrix} \right] \right)$ | (15) |
| --- | --- | --- |

We equally adapted the main model and the two additional models including “birth.registration” and “fragile.state” (Figure 4, Supplementary Figure 7). To avoid replication, we only show the formula for the main model:

|  | $outcome\sim\mathrm{Binomial}\left( 1, p_{ij} \right)$ | (16) |
| --- | --- | --- |

|  | $\mathrm{logit}\left( p_{ij} \right)=\beta_{0j}+\beta_{1j}\times year_{ij}+\beta_{2}\times vaccine_{ij}+\beta_{3j}\times year_{ij}\times vaccine_{ij}+$ $=\beta_{4}\times income_{i}+\beta_{5}\times region_{i}+\beta_{6}\times population. quintile_{i}+\beta_{7}\times gavi_{i}+\beta_{8}\times immunization.{coverage}_{i}$ | (17) |
| --- | --- | --- |

|  | $\left[ \begin{matrix} \beta_{0j} & \beta_{1j} & \beta_{2j} \end{matrix} \right]^{'}\sim\mathrm{MVN}\left( \left[ \begin{matrix} \gamma_{00} \\ \gamma_{10} \\ \gamma_{20} \end{matrix} \right],\left[ \begin{matrix} \tau_{0}^{2} & \phi_{01}\tau_{0}\tau_{1} & 0 \\ \phi_{01}\tau_{0}\tau_{1} & \tau_{1}^{2} & 0 \\ 0 & 0 & \tau_{2}^{2} \end{matrix} \right] \right)$ | (18) |
| --- | --- | --- |

## Software used

All analyses were conducted using the R language for statistical computing, version 4.0.2 [1]. The a priori significance level was set at p *=* 0.05. The glmmTMB package version 1.0.2.1 was used to fit mixed-effects models [2]. Predicted time trends for data quality flags were calculated using the emmeans package [3] version 1.5.2.1 and ggeffects package [4] version 0.16.0.

# Supplementary Tables

**Supplementary Table 1: Immunization coverage data points and reporting countries affected by potential data quality issues, by vaccine dose, 194 WHO Member States, 2000–2019.**

|  | **Data points affected/data points reported (%)** | |  | **Countries affected/countries reporting (%)** | |
| --- | --- | --- | --- | --- | --- |
| **Vaccine dose** | | | | | |
| MCV1 | 5802/14604 | (40%) |  | 188/194 | (97%) |
| BCG | 4965/12024 | (41%) |  | 151/152 | (99%) |
| DTP3 | 6977/14604 | (48%) |  | 189/194 | (97%) |
| DTP1 | 8646/14604 | (59%) |  | 193/194 | (99%) |

Notes: Country data as reported by 15 July 2020. BCG = Bacillus Calmette-Guérin vaccine birth dose. DTP1 = first dose of diphtheria-tetanus-pertussis-containing vaccine. DTP3 = third dose of diphtheria-tetanus-pertussis-containing vaccine. MCV1 = first dose of measles-containing vaccine.

**Supplementary Table 2:** **Modeled probability of data quality flags for immunization coverage reports for DTP1, DTP3, MCV1, and BCG globally and by different country classifications, 194 WHO Member States, 2000–2019.**

|  | **Probability of** | | |
| --- | --- | --- | --- |
|  | **data quality flags (%)** | | |
|  | Estimate | (95% CI) | p-value* |
| **Global** | | | |
| Overall | 18.2 | (14.8 – 22.3) | NA |
| **WHO World Region** | | | |
| African Region | 23.2 | (17.3 – 30.5) | **0.013** |
| Region of the Americas | 29.7 | (22.7 – 37.9) | **<0.001** |
| South-East Asia Region | 6.3 | (3.3 – 11.8) | **0.002** |
| European Region | 11.8 | (8.5 – 16.2) | 0.099 |
| Eastern Mediterranean Region | 14.3 | (9.5 – 21.0) | 0.61 |
| Western Pacific Region | 17.4 | (12 – 24.5) | 0.61 |
| **World Bank income group** | | | |
| High income | 17.1 | (12.5 – 23) | 0.56 |
| Upper-middle income | 12.8 | (9.8 – 16.4) | **0.033** |
| Lower-middle income | 15.9 | (12.9 – 19.4) | 0.83 |
| Low income | 17.2 | (13.4 – 21.9) | 0.56 |
| **Population size** | | | |
| Lowest quintile | 19.0 | (13.8 – 25.5) | 0.24 |
| Second quintile | 15.5 | (11.5 – 20.7) | 0.95 |
| Third quintile | 13.6 | (10.2 – 18.0) | 0.24 |
| Fourth quintile | 18.0 | (13.8 – 23.1) | 0.24 |
| Highest quintile | 12.8 | (9.6 – 17.0) | 0.24 |
| **Immunization coverage level** |  |  |  |
| Below 80% immunized | 16.6 | (11.4 – 23.4) | 0.9 |
| 80% to under 90% immunized | 20.1 | (14.5 – 27.3) | 0.096 |
| 90% to under 95% immunized | 11.2 | (8.1 – 15.2) | **0.024** |
| 95% and more immunized | 15.8 | (12.8 – 19.5) | 0.92 |
| **Birth registration level** |  |  |  |
| Below 80% registered | 13.7 | (9.3 – 19.9) | 0.81 |
| 80% to under 90% registered | 18.6 | (11.5 – 28.7) | 0.81 |
| 90% to under 95% registered | 13.5 | (7.9 – 22.1) | 0.81 |
| 95% and more registered | 14.6 | (10.8 – 19.4) | 0.88 |
| **Fragile and conflict-affected situations (FCS)** | | | |
| no | 12.0 | (9.4 – 15.1) | 0.12 |
| yes | 14.7 | (11.0 – 19.6) | 0.12 |
| **Support by Gavi, the Vaccine Alliance** | | | |
| no | 17.5 | (13.9 – 21.8) | 0.23 |
| yes | 14.0 | (10.4 – 18.5) | 0.23 |

Notes: * p-values < 0.05 were considered a statistically significant difference from the classification mean. Country data as reported by 15 July 2020. BCG = Bacillus Calmette-Guérin vaccine birth dose. CI = confidence interval. DTP1 = first dose of diphtheria-tetanus-pertussis-containing vaccine. DTP3 = third dose of diphtheria-tetanus-pertussis-containing vaccine. FCS = fragile and conflict-affected situations. MCV1 = first dose of measles-containing vaccine. NA = not applicable. Countries were grouped separately for each year by World Bank income groups, population size, and fragile and conflict-affected situations (FCS) classification. All other groupings were done for all years together. FCS status was available for 2004–2019 only. Immunization coverage level was based on average DTP1 and DTP3 coverage estimated by WHO and UNICEF for 2017–2019, as of July 2020. Birth registration levels refer to children under age five who have been registered based on the latest available UNICEF estimate. Support by Gavi, the Vaccine Alliance, refers to funding in any year between 2000 and 2019.

**Supplementary Table 3: Modeled probability of data quality flags for immunization coverage reports, by vaccine dose, 194 WHO Member States, 2000–2019.**

|  | **Probability of** | | |
| --- | --- | --- | --- |
|  | **data quality flags (%)** | | |
|  | Estimate | (95% CI) | p-value* |
| **Vaccine dose** | | | |
| BCG | 8.2 | (6.1 – 10.8) | **<0.001** |
| DTP1 | 36.0 | (29.8 – 42.6) | **<0.001** |
| DTP3 | 19.3 | (15.9 – 23.3) | **<0.001** |
| MCV1 | 9.0 | (7.1 – 11.5) | **<0.001** |

Notes: * p-values < 0.05 were considered a statistically significant difference from the mean. Country data as reported by 15 July 2020. BCG = Bacillus Calmette-Guérin vaccine birth dose. CI = confidence interval. DTP1 = first dose of diphtheria-tetanus-pertussis-containing vaccine. DTP3 = third dose of diphtheria-tetanus-pertussis-containing vaccine. MCV1 = first dose of measles-containing vaccine.

Supplementary Table 4: Modeled trends of the probability of data quality flags for immunization coverage reports for DTP1, DTP3, MCV1, and BCG, by different country classifications, 194 WHO Member States, 2000–2019.

|  | **Yearly trend of probability of** | | |
| --- | --- | --- | --- |
|  | **data quality flags (%)** | | |
|  | Coefficient | (95% CI) | p-value* |
| **Global** | | | |
| Overall | -5.1 | (-7.0 to -3.2) | **<0.001** |
| **WHO World Region** | | | |
| African Region | -9.6 | (-13.0 to -5.8) | **<0.001** |
| Region of the Americas | -4.9 | (-9.2 to -0.6) | **0.026** |
| South-East Asia Region | 3.1 | (-5.2 to 11.0) | 0.46 |
| European Region | -5.4 | (-9.2 to -1.6) | **0.0055** |
| Eastern Mediterranean Region | -0.054 | (-5.7 to 5.5) | 0.98 |
| Western Pacific Region | -5.1 | (-10.0 to 0.13) | 0.056 |
| **World Bank income group** | | | |
| High income | -8.4 | (-12.0 to -4.8) | **<0.001** |
| Upper-middle income | -3.5 | (-6.7 to -0.37) | **0.029** |
| Lower-middle income | -1.4 | (-4.5 to 1.7) | 0.36 |
| Low income | -8.2 | (-12.0 to -4.7) | **<0.001** |
| **Population size** | | | |
| Lowest quintile | -6.9 | (-11.0 to -2.6) | **0.0016** |
| Second quintile | -8.0 | (-12.0 to -3.9) | **<0.001** |
| Third quintile | -5.0 | (-8.9 to -1.0) | **0.013** |
| Fourth quintile | -5.4 | (-9.1 to -1.6) | **0.0052** |
| Highest quintile | -1.4 | (-5.6 to 2.7) | 0.49 |
| **Immunization coverage level** |  |  |  |
| Below 80% immunized | -3.3 | (-8.2 to 1.6) | 0.18 |
| 80% to under 90% immunized | -8.8 | (-13.0 to -4.1) | **<0.001** |
| 90% to under 95% immunized | -8.0 | (-12.0 to -3.7) | **<0.001** |
| 95% and more immunized | -3.6 | (-6.5 to -0.79) | **0.012** |
| **Birth registration level** |  |  |  |
| Below 80% registered | -6.9 | (-11.0 to -2.8) | **0.001** |
| 80% to under 90% registered | -5.4 | (-12.0 to 0.92) | 0.094 |
| 90% to under 95% registered | -1.3 | (-8.5 to 5.9) | 0.73 |
| 95% and more registered | -4.4 | (-7.3 to -1.6) | **0.002** |
| **Fragile and conflict-affected situations (FCS)** | | | |
| no | -5.1 | (-8.1 to -2.1) | **<0.001** |
| yes | -5.2 | (-10.0 to 0.002) | 0.05 |
| **Support by Gavi, the Vaccine Alliance** | | | |
| no | -4.4 | (-7.0 to -1.8) | **<0.001** |
| yes | -6.9 | (-10.0 to -3.7) | **<0.001** |

Notes: * p-values < 0.05 were considered a statistically significant trend (increasing or decreasing). Country data as reported by 15 July 2020. BCG = Bacillus Calmette-Guérin vaccine birth dose. CI = confidence interval. DTP1 = first dose of diphtheria-tetanus-pertussis-containing vaccine. DTP3 = third dose of diphtheria-tetanus-pertussis-containing vaccine. FCS = fragile and conflict-affected situations. MCV1 = first dose of measles-containing vaccine. Countries were grouped separately for each year by World Bank income groups, population size, and fragile and conflict-affected situations (FCS) classification. All other groupings were done for all years together. FCS status was available for 2004–2019 only. Immunization coverage level was based on average DTP1 and DTP3 coverage estimated by WHO and UNICEF for 2017–2019, as of July 2020. Birth registration levels refer to children under age five who have been registered based on the latest available UNICEF estimate. Support by Gavi, the Vaccine Alliance, refers to funding in any year between 2000 and 2019.

**Supplementary Table 5: Modeled trends of the probability of data quality flags for immunization coverage reports, by vaccine dose, 194 WHO Member States, 2000–2019.**

|  | **Yearly trend of probability of** | | |
| --- | --- | --- | --- |
|  | **data quality flags (%)** | | |
|  | Coefficient | (95% CI) | p-value* |
| **Vaccine dose** | | | |
| BCG | -4.8 | (-7.6 to -2.0) | **<0.001** |
| DTP1 | -6.7 | (-9.1 to -4.4) | **<0.001** |
| DTP3 | -4.7 | (-7.1 to -2.3) | **<0.001** |
| MCV1 | -4.3 | (-6.9 to -1.7) | **0.001** |

Notes: * p-values of < 0.05 were considered a statistically significant trend (increasing or decreasing). Country data as reported by 15 July 2020. BCG = Bacillus Calmette-Guérin vaccine birth dose. CI = confidence interval. DTP1 = first dose of diphtheria-tetanus-pertussis-containing vaccine. DTP3 = third dose of diphtheria-tetanus-pertussis-containing vaccine. MCV1 = first dose of measles-containing vaccine.

# Supplementary Figures

**Supplementary Figure 1: Method for flagging the quality of reported BCG immunization data by type of country immunization reporting system for 152 WHO Member States.**

Notes: BCG = Bacillus Calmette–Guérin vaccine birth dose. IMR = infant mortality rate (denominator for BCG – denominator for DTP1 or DTP3 or MCV1 / denominator for BCG). UI = uncertainty interval. UN-IGME = United Nations Inter-agency Group for Child Mortality Estimation. UNPD = United Nations Population Division.

no

yes

yes

yes

yes

no

- Denominator missing
- Implied IMR zero or negative
- BCG denominator versus UNPD live births ≥10%
- Implied IMR outside 90% UI of UN-IGME IMR
- Same denominator as in preceding year
- Denominator year–to–year difference
  ≥10%

or

or

- Numerator missing
- Recalculated admin coverage ≥100%
- Same numerator as in preceding year
- Admin coverage missing
- Admin coverage ≥100%
- Admin coverage year–to–year difference
  ≥10%
- Admin coverage different from recalculated admin coverage

Countries with BCG birth dose and centralized immunization reporting system that report admin and official coverage data (n = 151)

Country–year–BCG coverage
report flagged

Country–year–BCG coverage
report not flagged

- Official coverage missing
- Official coverage year–to–year difference
  ≥10%
- Official coverage ≥100%

Countries with BCG birth dose and without centralized immunization reporting system that report official coverage data only (n = 1)

**Supplementary Figure 2: Method for flagging the quality of reported DTP1 immunization data by type of country immunization reporting system for 194 WHO Member States.**

Notes: DTP1 = First dose of diphtheria–tetanus–pertussis-containing vaccine. DTP3 = Third dose of diphtheria–tetanus–pertussis-containing vaccine. UNPD = United Nations Population Division.

no

yes

yes

no

yes

- Official coverage missing
- Official coverage year–to–year difference
  ≥10%
- Official coverage ≥100%
- Negative DTP3 to DTP1 official coverage dropout rate

or

or

- Numerator missing
- Recalculated admin coverage ≥100%
- Same numerator as in preceding year
- Negative DTP3 to DTP1 doses dropout rate
- Denominator missing
- DTP1 denominator versus UNPD surviving infants ≥10%
- Same denominator as in preceding year
- Denominator year–to–year difference
  ≥10%
- Admin coverage missing
- Admin coverage ≥100%
- Admin coverage year–to–year difference ≥10%
- Admin coverage different from recalculated admin coverage
- Negative DTP3 to DTP1 admin coverage dropout rate

yes

Countries with centralized immunization reporting system that report admin and official coverage data (n = 180)

Country–year–DTP1 coverage
report flagged

Country–year–DTP1 coverage
report not flagged

Countries without centralized immunization reporting system that report official coverage
data only (n = 14)

no

yes

yes

no

yes

- Official coverage missing
- Official coverage year–to–year difference ≥10%
- Official coverage ≥100%
- Negative DTP3 to DTP1 official coverage dropout rate

or

or

- Numerator missing
- Recalculated admin coverage ≥100%
- Same numerator as in preceding year
- Negative DTP3 to DTP1 doses dropout rate
- Denominator missing
- DTP3 denominator versus UNPD surviving infants ≥10%
- Same denominator as in preceding year
- Denominator year–to–year difference
  ≥10%
- Admin coverage missing
- Admin coverage ≥100%
- Admin coverage year–to–year difference ≥10%
- Admin coverage different from recalculated admin coverage
- Negative DTP3 to DTP1 admin coverage dropout rate

yes

Countries with centralized immunization reporting system that report admin and official coverage data (n = 180)

Country–year–DTP3 coverage
report flagged

Country–year–DTP3 coverage
report not flagged

Countries without centralized immunization reporting system that report official coverage
data only (n = 14)

**Supplementary Figure 3: Method for flagging the quality of reported DTP3 immunization data by type of country immunization reporting system for 194 WHO Member States.**

Notes: DTP1 = First dose of diphtheria–tetanus–pertussis-containing vaccine. DTP3 = Third dose of diphtheria–tetanus–pertussis-containing vaccine. UNPD = United Nations Population Division.

**Supplementary Figure 4: Method for flagging the quality of MCV1 immunization data by type of country immunization reporting system for 194 WHO Member States.**

Notes: MCV1 = First dose of measles-containing vaccine. UNPD = United Nations Population Division.

yes

- Denominator missing
- Same denominator as in preceding year
- Denominator year–to–year difference
  ≥10%
- MCV1 denominator vs. UNPD surviving infants ≥10%

yes

yes

yes

no

or

or

- Numerator missing
- Recalculated admin coverage ≥100%
- Same numerator as in preceding year
- Admin coverage missing
- Admin coverage ≥100%
- Admin coverage year–to–year difference ≥10%
- Admin coverage different from recalculated admin coverage

Countries with centralized immunization reporting system that report admin and official coverage data (n = 180)

Country–year–MCV1 coverage
report flagged

Country–year–MCV1 coverage
report not flagged

- Official coverage missing
- Official coverage year–to–year difference
  ≥10%
- Official coverage ≥100%

Countries without centralized immunization reporting system that report official coverage
data only (n = 14)

no


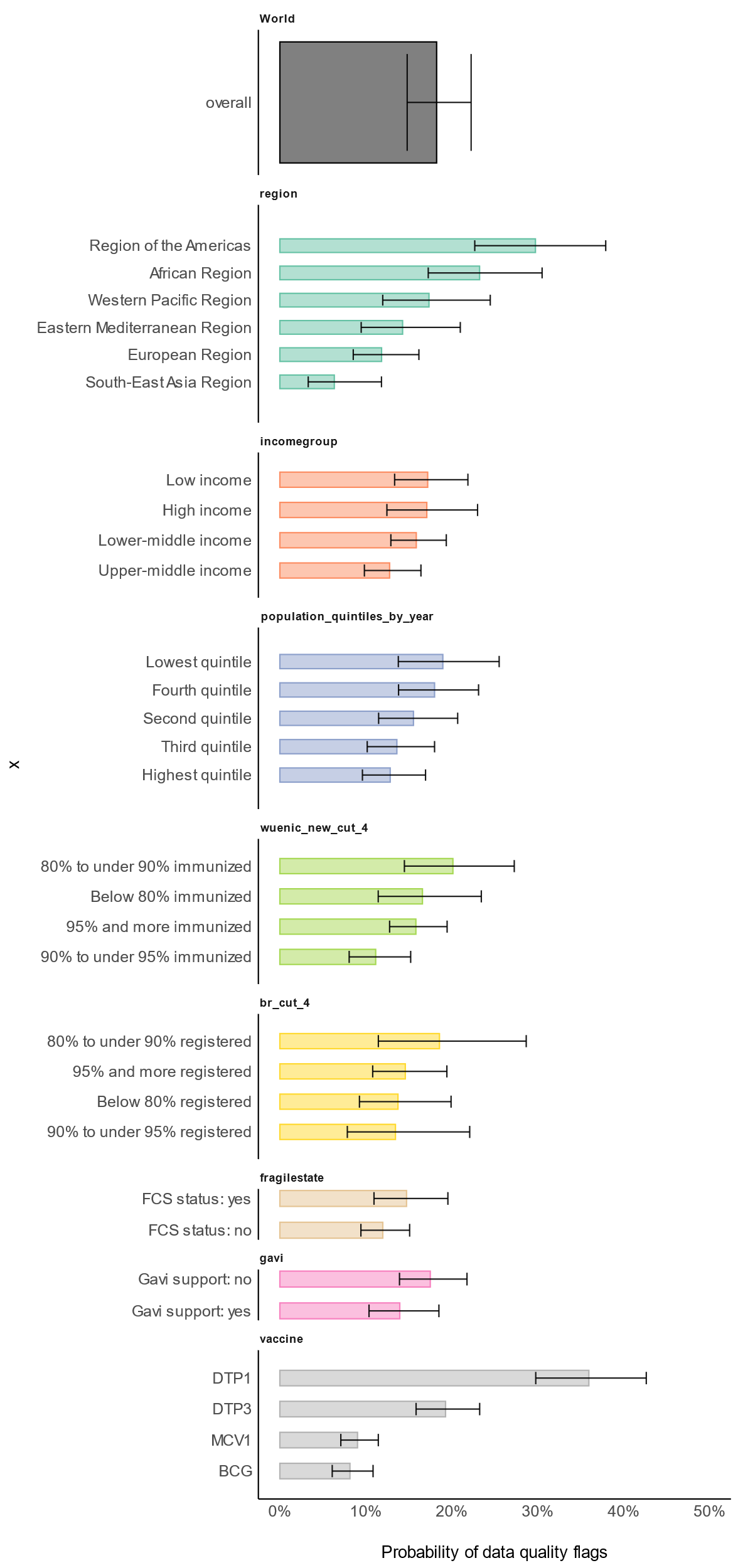


**Supplementary Figure 5: Modeled probability of data quality flags for immunization coverage reports, by vaccine dose, 194 WHO Member States, 2000–2019.**

Notes: Error bars represent 95% confidence intervals (CI). Country data as reported by 15 July 2020. BCG = Bacillus Calmette-Guérin vaccine birth dose. DTP1 = first dose of diphtheria-tetanus-pertussis-containing vaccine. DTP3 = third dose of diphtheria-tetanus-pertussis-containing vaccine. MCV1 = first dose of measles-containing vaccine.

-15%

-10%

-5%

0%

5%

10%

15%

DTP1

BCG

DTP3

MCV1

Trend of probability of data quality flags

**Supplementary Figure 6: Modeled trends of the probability of data quality flags for immunization coverage reports, by vaccine dose, 194 WHO Member States, 2000–2019.**

Notes: Lines represent 95% confidence intervals (CI). Country data as reported by 15 July 2020. BCG = Bacillus Calmette-Guérin vaccine birth dose. DTP1 = first dose of diphtheria-tetanus-pertussis-containing vaccine. DTP3 = third dose of diphtheria-tetanus-pertussis-containing vaccine. MCV1 = first dose of measles-containing vaccine.

**D**

**T**

**P**

**3**

**M**

**C**

**V**

**1**

**B**

**C**

**G**

**D**

**T**

**P**

**1**

2

0

0

0

2

0

0

5

2

0

1

0

2

0

1

5

2

0

0

0

2

0

0

5

2

0

1

0

2

0

1

5

0

%

2

5

%

5

0

%

7

5

%

1

0

0

%

0

%

2

5

%

5

0

%

7

5

%

1

0

0

%

Y

e

a

r

P

r

o

b

a

b

i

l

i

t

y

o

f

d

a

t

a

q

u

a

l

i

t

y

f

l

a

g

s

**Supplementary Figure 7: Modeled trends of the probability of data quality flags for immunization coverage reports for DTP1, DTP3, MCV1, and BCG, by vaccine dose, 194 WHO Member States, 2000–2019.**

Notes: Shading represents 95% confidence intervals (CI). Country data as reported by 15 July 2020. BCG = Bacillus Calmette-Guérin vaccine birth dose. DTP1 = first dose of diphtheria-tetanus-pertussis-containing vaccine. DTP3 = third dose of diphtheria-tetanus-pertussis-containing vaccine. MCV1 = first dose of measles-containing vaccine.

# Lists of countries

## WHO Member States

Afghanistan, Albania, Algeria, Andorra, Angola, Antigua and Barbuda, Argentina, Armenia, Australia, Austria, Azerbaijan, Bahamas, Bahrain, Bangladesh, Barbados, Belarus, Belgium, Belize, Benin, Bhutan, Bolivia (Plurinational State of), Bosnia and Herzegovina, Botswana, Brazil, Brunei Darussalam, Bulgaria, Burkina Faso, Burundi, Cabo Verde, Cambodia, Cameroon, Canada, Central African Republic, Chad, Chile, China, Colombia, Comoros, Congo, Cook Islands, Costa Rica, Côte d'Ivoire, Croatia, Cuba, Cyprus, Czechia, Democratic People's Republic of Korea, Democratic Republic of the Congo, Denmark, Djibouti, Dominica, Dominican Republic, Ecuador, Egypt, El Salvador, Equatorial Guinea, Eritrea, Estonia, Eswatini, Ethiopia, Fiji, Finland, France, Gabon, Gambia, Georgia, Germany, Ghana, Greece, Grenada, Guatemala, Guinea, Guinea-Bissau, Guyana, Haiti, Honduras, Hungary, Iceland, India, Indonesia, Iran (Islamic Republic of), Iraq, Ireland, Israel, Italy, Jamaica, Japan, Jordan, Kazakhstan, Kenya, Kiribati, Kuwait, Kyrgyzstan, Lao People's Democratic Republic, Latvia, Lebanon, Lesotho, Liberia, Libya, Lithuania, Luxembourg, Madagascar, Malawi, Malaysia, Maldives, Mali, Malta, Marshall Islands, Mauritania, Mauritius, Mexico, Micronesia (Federated States of), Monaco, Mongolia, Montenegro, Morocco, Mozambique, Myanmar, Namibia, Nauru, Nepal, Netherlands, New Zealand, Nicaragua, Niger, Nigeria, Niue, North Macedonia, Norway, Oman, Pakistan, Palau, Panama, Papua New Guinea, Paraguay, Peru, Philippines, Poland, Portugal, Qatar, Republic of Korea, Republic of Moldova, Romania, Russian Federation, Rwanda, Saint Kitts and Nevis, Saint Lucia, Saint Vincent and the Grenadines, Samoa, San Marino, Sao Tome and Principe, Saudi Arabia, Senegal, Serbia, Seychelles, Sierra Leone, Singapore, Slovakia, Slovenia, Solomon Islands, Somalia, South Africa, South Sudan, Spain, Sri Lanka, Sudan, Suriname, Sweden, Switzerland, Syrian Arab Republic, Tajikistan, Thailand, Timor-Leste, Togo, Tonga, Trinidad and Tobago, Tunisia, Turkey, Turkmenistan, Tuvalu, Uganda, Ukraine, United Arab Emirates, United Kingdom of Great Britain and Northern Ireland, United Republic of Tanzania, United States of America, Uruguay, Uzbekistan, Vanuatu, Venezuela (Bolivarian Republic of), Viet Nam, Yemen, Zambia, Zimbabwe

## WHO World Region

WHO groups its Member States according to regional distribution into six groups (African Region, Region of the Americas, South-East Asia Region, European Region, Eastern Mediterranean Region, Western Pacific Region). Regional groupings for all WHO Member States were retrieved from WHO, as of July 2020 [5].

**WHO African Region – AFR (47 countries)**

Algeria, Angola, Benin, Botswana, Burkina Faso, Burundi, Cabo Verde, Cameroon, Central African Republic, Chad, Comoros, Congo, Côte d’Ivoire, Democratic Republic of the Congo, Equatorial Guinea, Eritrea, Eswatini, Ethiopia, Gabon, Gambia, Ghana, Guinea, Guinea-Bissau, Kenya, Lesotho, Liberia, Madagascar, Malawi, Mali, Mauritania, Mauritius, Mozambique, Namibia, Niger, Nigeria, Rwanda, Sao Tome and Principe, Senegal, Seychelles, Sierra Leone, South Africa, South Sudan, Togo, Uganda, United Republic of Tanzania, Zambia, Zimbabwe

**WHO World Region of the Americas – AMR (35 countries)**

Antigua and Barbuda, Argentina, Bahamas, Barbados, Belize, Bolivia (Plurinational State of), Brazil, Canada, Chile, Colombia, Costa Rica, Cuba, Dominica, Dominican Republic, Ecuador, El Salvador, Grenada, Guatemala, Guyana, Haiti, Honduras, Jamaica, Mexico, Nicaragua, Panama, Paraguay, Peru, Saint Kitts and Nevis, Saint Lucia, Saint Vincent and the Grenadines, Suriname, Trinidad and Tobago, the United States of America, Uruguay, Venezuela (Bolivarian Republic of)

**WHO South-East Asia Region – SEAR (11 countries)**

Bangladesh, Bhutan, Democratic People’s Republic of Korea, India, Indonesia, Maldives, Myanmar, Nepal, Sri Lanka, Thailand, Timor-Leste

**WHO European Region – EUR (53 countries)**

Albania, Andorra, Armenia, Austria, Azerbaijan, Belarus, Belgium, Bosnia and Herzegovina, Bulgaria, Croatia, Cyprus, Czechia, Denmark, Estonia, Finland, France, Georgia, Germany, Greece, Hungary, Iceland, Ireland, Israel, Italy, Kazakhstan, Kyrgyzstan, Latvia, Lithuania, Luxembourg, Malta, Monaco, Montenegro, Netherlands, North Macedonia, Norway, Poland, Portugal, Republic of Moldova, Romania, Russian Federation, San Marino, Serbia, Slovakia, Slovenia, Spain, Sweden, Switzerland, Tajikistan, Turkey, Turkmenistan, Ukraine, United Kingdom of Great Britain and Northern Ireland, Uzbekistan

**WHO Eastern Mediterranean Region – EMR (21 countries)**

Afghanistan, Bahrain, Djibouti, Egypt, Iran (Islamic Republic of), Iraq, Jordan, Kuwait, Lebanon, Libya, Morocco, Oman, Pakistan, Qatar, Saudi Arabia, Somalia, Sudan, Syrian Arab Republic, Tunisia, United Arab Emirates, Yemen

**WHO Western Pacific Region – WPR (27 countries)**

Australia, Brunei Darussalam, Cambodia, China, Cook Islands, Fiji, Japan, Kiribati, Lao People’s Democratic Republic, Malaysia, Marshall Islands, Micronesia (Federated States of), Mongolia, Nauru, New Zealand, Niue, Palau, Papua New Guinea, Philippines, Republic of Korea, Samoa, Singapore, Solomon Islands, Tonga, Tuvalu, Vanuatu, Viet Nam

## World Bank income group

The World Bank groups its Member States into four income groups based on gross national income (GNI) per capita in United States dollars (high income, upper-middle income, lower-middle income, low income). Historical classifications on country groups for each year between 2000 and 2019 for 192 WHO Member States were retrieved from the World Bank, as of July 2020 [6]. The following table shows the income ranges used for this classification:

**Income ranges for the classification of World Bank Member States by year, 2000–2019, in US$.**

| **Year** | **Low income** | **Lower-middle income** | **Upper-middle income** | | **High income** | |
| --- | --- | --- | --- | --- | --- | --- |
| 2000 | ≤ 755 | 756 – 2,995 | | 2,996 – 9,265 | | > 9,265 |
| 2001 | ≤ 745 | 746 – 2,975 | | 2,976 – 9,205 | | > 9,205 |
| 2002 | ≤ 735 | 736 – 2,935 | | 2,936 – 9,075 | | > 9,075 |
| 2003 | ≤ 765 | 766 – 3,035 | | 3,036 – 9,385 | | > 9,385 |
| 2004 | ≤ 825 | 826 – 3,255 | | 3,256 – 10,065 | | > 10,065 |
| 2005 | ≤ 875 | 876 – 3,465 | | 3,466 – 10,725 | | > 10,725 |
| 2006 | ≤ 905 | 906 – 3,595 | | 3,596 – 11,115 | | > 11,115 |
| 2007 | ≤ 935 | 936 – 3,705 | | 3,706 – 11,455 | | > 11,455 |
| 2008 | ≤ 975 | 976 – 3,855 | | 3,856 – 11,905 | | > 11,905 |
| 2009 | ≤ 995 | 996 – 3,945 | | 3,946 – 12,195 | | > 12,195 |
| 2010 | ≤ 1,005 | 1,006 – 3,975 | | 3,976 – 12,275 | | > 12,275 |
| 2011 | ≤ 1,025 | 1,026 – 4,035 | | 4,036 – 12,475 | | > 12,475 |
| 2012 | ≤ 1,035 | 1,036 – 4,085 | | 4,086 – 12,615 | | > 12,615 |
| 2013 | ≤ 1,045 | 1,046 – 4,125 | | 4,126 – 12,745 | | > 12,745 |
| 2014 | ≤ 1,045 | 1,046 – 4,125 | | 4,126 – 12,735 | | > 12,735 |
| 2015 | ≤ 1,025 | 1,026 – 4,035 | | 4,036 – 12,475 | | > 12,475 |
| 2016 | ≤ 1,005 | 1,006 – 3,955 | | 3,956 – 12,235 | | > 12,235 |
| 2017 | ≤ 995 | 996 – 3,895 | | 3,896 – 12,055 | | > 12,055 |
| 2018 | ≤ 1,025 | 1,026 – 3,995 | | 3,996 – 12,375 | | > 12,375 |
| 2019 | ≤ 1,035 | 1,036 – 4,045 | | 4,046 – 12,535 | | > 12,535 |
|  |  |  | |  | |  |

**High income (64 countries)**

Andorra, Antigua and Barbuda (2002, 2005-2008, 2012-2019), Argentina (2014, 2017), Australia, Austria, Bahamas, Bahrain (2001-2019), Barbados (2000, 2002, 2006-2019), Belgium, Brunei Darussalam, Canada, Chile (2012-2019), Croatia (2008-2015, 2017-2019), Cyprus, Czechia (2006-2019), Denmark, Equatorial Guinea (2007-2014), Estonia (2006-2019), Finland, France, Germany, Greece, Hungary (2007-2011, 2014-2019), Iceland, Ireland, Israel, Italy, Japan, Kuwait, Latvia (2009, 2012-2019), Lithuania (2012-2019), Luxembourg, Malta (2000, 2002-2019), Mauritius (2019), Monaco, Nauru (2015, 2019), Netherlands, New Zealand, Norway, Oman (2007-2019), Palau (2016-2019), Panama (2017-2019), Poland (2009-2019), Portugal, Qatar, Republic of Korea (2001-2019), Romania (2019), Russian Federation (2012-2014), Saint Kitts and Nevis (2011-2019), San Marino, Saudi Arabia (2004-2019), Seychelles (2014-2019), Singapore, Slovakia (2007-2019), Slovenia, Spain, Sweden, Switzerland, Trinidad and Tobago (2006-2019), United Arab Emirates, United Kingdom of Great Britain and Northern Ireland, United States of America, Uruguay (2012-2019), Venezuela (Bolivarian Republic of; 2014)

**Upper-middle income (84 countries)**

Albania (2009-2010, 2012-2019), Algeria (2008-2018), Angola (2011-2015), Antigua and Barbuda (2000-2001, 2003-2004, 2009-2011), Argentina (2000-2013, 2015-2016, 2018-2019), Armenia (2017-2019), Azerbaijan (2009-2019), Bahrain (2000), Barbados (2001, 2003-2005), Belarus (2007-2019), Belize (2002-2007, 2012-2019), Bosnia and Herzegovina (2008-2019), Botswana, Brazil (2000-2001, 2006-2019), Bulgaria (2006-2019), Chile (2000-2011), China (2010-2019), Colombia (2008-2019), Costa Rica, Croatia (2000-2007, 2016), Cuba (2007-2019), Czechia (2000-2005), Dominica, Dominican Republic (2008-2019), Ecuador (2010-2019), Equatorial Guinea (2004-2006, 2015-2019), Estonia (2000-2005), Fiji (2007-2009, 2012-2019), Gabon, Georgia (2015, 2018-2019), Grenada, Guatemala (2017-2019), Guyana (2015-2019), Hungary (2000-2006, 2012-2013), Indonesia (2019), Iran (Islamic Republic of; 2009-2019), Iraq (2012-2019), Jamaica (2007-2019), Jordan (2010-2015, 2017-2019), Kazakhstan (2006-2019), Latvia (2001-2008, 2010-2011), Lebanon, Libya, Lithuania (2001-2011), Malaysia, Maldives (2010-2019), Malta (2001), Marshall Islands (2012-2019), Mauritius (2000-2018), Mexico, Mongolia (2014), Montenegro (2006-2019), Namibia (2008-2019), Nauru (2016-2018), North Macedonia (2008-2019), Oman (2000-2006), Palau (2000-2015), Panama (2000-2016), Paraguay (2014-2019), Peru (2008-2019), Poland (2000-2008), Republic of Korea (2000), Romania (2005-2018), Russian Federation (2004-2019), Saint Kitts and Nevis (2000-2010), Saint Lucia, Saint Vincent and the Grenadines (2003-2019), Samoa (2016-2019), Saudi Arabia (2000-2003), Serbia (2006-2019), Seychelles (2000-2013), Slovakia (2000-2006), South Africa (2000, 2004-2019), Sri Lanka (2018), Suriname (2007-2019), Thailand (2010-2019), Tonga (2012-2014, 2016-2019), Trinidad and Tobago (2000-2005), Tunisia (2010-2014), Turkey (2000, 2004-2019), Turkmenistan (2011-2019), Tuvalu (2011-2019), Uruguay (2000-2011), Venezuela (Bolivarian Republic of; 2000-2013, 2015-2019)

**Lower-middle income (97 countries)**

Albania (2000-2008, 2011), Algeria (2000-2007, 2019), Angola (2004-2010, 2016-2019), Armenia (2002-2016), Azerbaijan (2003-2008), Bangladesh (2014-2019), Belarus (2000-2006), Belize (2000-2001, 2008-2011), Benin (2019), Bhutan (2006-2019), Bolivia (Plurinational State of), Bosnia and Herzegovina (2000-2007), Brazil (2002-2005), Bulgaria (2000-2005), Côte d'Ivoire (2008-2019), Cabo Verde, Cambodia (2015-2019), Cameroon (2005-2019), China (2000-2009), Colombia (2000-2007), Comoros (2018-2019), Congo (2005-2019), Cuba (2000-2006), Djibouti, Dominican Republic (2000-2007), Ecuador (2000-2009), Egypt, El Salvador, Equatorial Guinea (2000), Eswatini, Fiji (2000-2006, 2010-2011), Georgia (2003-2017), Ghana (2010-2019), Guatemala (2000-2016), Guyana (2000-2014), Honduras, India (2007-2019), Indonesia (2003-2018), Iran (Islamic Republic of; 2000-2008), Iraq (2000-2011), Jamaica (2000-2006), Jordan (2000-2009, 2016), Kazakhstan (2000-2005), Kenya (2014-2019), Kiribati, Kyrgyzstan (2013-2019), Lao People's Democratic Republic (2010-2019), Latvia (2000), Lesotho (2005-2019), Lithuania (2000), Maldives (2000-2009), Marshall Islands (2000-2011), Mauritania (2010, 2012-2019), Micronesia (Federated States of), Mongolia (2007-2013, 2015-2019), Morocco, Myanmar (2014-2019), Namibia (2000-2007), Nepal (2019), Nicaragua (2005-2019), Nigeria (2008-2019), North Macedonia (2000-2007), Pakistan (2008-2019), Papua New Guinea (2000, 2008-2019), Paraguay (2000-2013), Peru (2000-2007), Philippines, Republic of Moldova (2005-2019), Romania (2000-2004), Russian Federation (2000-2003), Saint Vincent and the Grenadines (2000-2002), Samoa (2000-2015), Sao Tome and Principe (2008-2019), Senegal (2009-2014, 2018-2019), Solomon Islands (2008, 2010-2019), South Africa (2001-2003), South Sudan (2011, 2013), Sri Lanka (2000-2017, 2019), Sudan (2007-2018), Suriname (2000-2006), Syrian Arab Republic (2000-2016), Tajikistan (2014-2016), Thailand (2000-2009), Timor-Leste (2007-2019), Tonga (2000-2011, 2015), Tunisia (2000-2019, 2015-2019), Turkey (2001-2003), Turkmenistan (2000-2009, 2010), Tuvalu (2009-2010), Ukraine (2002-2019), United Republic of Tanzania (2019), Uzbekistan (2009-2019), Vanuatu, Viet Nam (2009-2019), Yemen (2009-2016), Zambia (2010-2019), Zimbabwe (2018-2019)

**Low income (68 countries)**

Afghanistan, Angola (2000-2003), Armenia (2000-2001), Azerbaijan (2000-2002), Bangladesh (2000-2013), Benin (2000-2018), Bhutan (2000-2005), Burkina Faso, Burundi, Côte d'Ivoire (2000-2007), Cambodia (2000-2014), Cameroon (2000-2004), Central African Republic, Chad, Comoros (2000-2017), Congo (2000-2004), Democratic People's Republic of Korea, Democratic Republic of the Congo, Equatorial Guinea (2001-2003), Eritrea, Ethiopia, Gambia, Georgia (2000-2002), Ghana (2000-2009), Guinea, Guinea-Bissau, Haiti, India (2000-2006), Indonesia (2000-2002), Kenya (2000-2013), Kyrgyzstan (2000-2012), Lao People's Democratic Republic (2000-2009), Lesotho (2000-2004), Liberia, Madagascar, Malawi, Mali, Mauritania (2000-2009, 2011), Mongolia (2000-2006), Mozambique, Myanmar (2000-2013), Nepal (2000-2018), Nicaragua (2000-2004), Niger, Nigeria (2000-2007), Pakistan (2000-2007), Papua New Guinea (2001-2007), Republic of Moldova (2000-2004), Rwanda, Sao Tome and Principe (2000-2007), Senegal (2000-2007, 2015-2017), Sierra Leone, Solomon Islands (2000-2007, 2009), Somalia, South Sudan (2012, 2014-2019), Sudan (2000-2006, 2019), Syrian Arab Republic (2017-2019), Tajikistan (2000-2013, 2017-2019), Timor-Leste (2001-2006), Togo, Uganda, Ukraine (2000-2001), United Republic of Tanzania (2000-2018), Uzbekistan (2000-2008), Viet Nam (2000-2008), Yemen (2000-2008, 2017-2019), Zambia (2000-2009), Zimbabwe (2000-2017)

## Population size

Countries were classified into quintiles of population size for each year between 2000 and 2019 using estimates of total population retrieved from the United Nations Population Division [7]. The following table shows the population size thresholds used for this classification.

**Thresholds for the classification of countries by quintile of total population size by year, 2000–2019.**

| **Year** | **20%** | **40%** | **60%** | **80%** | |  |
| --- | --- | --- | --- | --- | --- | --- |
| 2000 | 717,584.0 | 4,202,658.0 | 9,871,632.0 | | 24,769,955.0 | |
| 2001 | 733,015.0 | 4,187,093.0 | 10,036,104.0 | | 25,108,563.0 | |
| 2002 | 745,219.6 | 4,179,820.0 | 10,048,870.2 | | 25,378,349.0 | |
| 2003 | 763,454.4 | 4,199,536.0 | 10,062,916.0 | | 25,934,261.8 | |
| 2004 | 780,854.4 | 4,225,419.4 | 10,123,937.8 | | 26,719,805.2 | |
| 2005 | 830,366.4 | 4,322,524.4 | 10,197,608.6 | | 27,532,124.8 | |
| 2006 | 807,885.8 | 4,365,704.0 | 10,330,316.0 | | 27,840,483.2 | |
| 2007 | 827,488.2 | 4,396,187.6 | 10,400,659.0 | | 28,164,330.4 | |
| 2008 | 851,552.6 | 4,453,674.4 | 10,459,275.6 | | 28,491,688.6 | |
| 2009 | 872,821.8 | 4,515,507.2 | 10,541,366.4 | | 28,900,959.2 | |
| 2010 | 893,346.4 | 4,572,766.6 | 10,603,895.2 | | 29,519,388.4 | |
| 2011 | 893,346.4 | 4,572,766.6 | 10,603,895.2 | | 29,519,388.4 | |
| 2012 | 965,969.4 | 4,753,141.8 | 10,637,883.4 | | 31,452,830.0 | |
| 2013 | 999,165.0 | 4,809,576.6 | 10,797,330.4 | | 32,624,573.4 | |
| 2014 | 1,016,489.4 | 4,864,769.8 | 10,991,864.2 | | 33,699,419.2 | |
| 2015 | 1,028,023.6 | 4,924,843.2 | 11,117,905.2 | | 34,513,603.0 | |
| 2016 | 1,040,043.0 | 5,034,986.6 | 11,249,518.8 | | 35,228,982.6 | |
| 2017 | 1,052,522.6 | 5,147,821.2 | 11,309,974.2 | | 35,867,198.2 | |
| 2018 | 1,065,337.8 | 5,263,079.6 | 11,456,370.8 | | 36,447,280.6 | |
| 2019 | 1,078,302.0 | 5,379,187.2 | 11,537,578.4 | | 36,847,480.2 | |

**Countries with UNPD estimate of total population of < 90,000 people in the year 2019 (11 countries)**

Andorra, Cook Islands, Dominica, Marshall Islands, Monaco, Nauru, Niue, Palau, Saint Kitts and Nevis, San Marino, Tuvalu

**Countries with UNPD estimate of total population of 90,000 or more people in the year 2019 (183 countries)**

Afghanistan, Albania, Algeria, Angola, Antigua and Barbuda, Argentina, Armenia, Australia, Austria, Azerbaijan, Bahamas, Bahrain, Bangladesh, Barbados, Belarus, Belgium, Belize, Benin, Bhutan, Bolivia (Plurinational State of), Bosnia and Herzegovina, Botswana, Brazil, Brunei Darussalam, Bulgaria, Burkina Faso, Burundi, Cabo Verde, Cambodia, Cameroon, Canada, Central African Republic, Chad, Chile, China, Colombia, Comoros, Congo, Costa Rica, Côte d'Ivoire, Croatia, Cuba, Cyprus, Czechia, Democratic People's Republic of Korea, Democratic Republic of the Congo, Denmark, Djibouti, Dominican Republic, Ecuador, Egypt, El Salvador, Equatorial Guinea, Eritrea, Estonia, Eswatini, Ethiopia, Fiji, Finland, France, Gabon, Gambia, Georgia, Germany, Ghana, Greece, Grenada, Guatemala, Guinea, Guinea-Bissau, Guyana, Haiti, Honduras, Hungary, Iceland, India, Indonesia, Iran (Islamic Republic of), Iraq, Ireland, Israel, Italy, Jamaica, Japan, Jordan, Kazakhstan, Kenya, Kiribati, Kuwait, Kyrgyzstan, Lao People's Democratic Republic, Latvia, Lebanon, Lesotho, Liberia, Libya, Lithuania, Luxembourg, Madagascar, Malawi, Malaysia, Maldives, Mali, Malta, Mauritania, Mauritius, Mexico, Micronesia (Federated States of), Mongolia, Montenegro, Morocco, Mozambique, Myanmar, Namibia, Nepal, Netherlands, New Zealand, Nicaragua, Niger, Nigeria, North Macedonia, Norway, Oman, Pakistan, Panama, Papua New Guinea, Paraguay, Peru, Philippines, Poland, Portugal, Qatar, Republic of Korea, Republic of Moldova, Romania, Russian Federation, Rwanda, Saint Lucia, Saint Vincent and the Grenadines, Samoa, Sao Tome and Principe, Saudi Arabia, Senegal, Serbia, Seychelles, Sierra Leone, Singapore, Slovakia, Slovenia, Solomon Islands, Somalia, South Africa, South Sudan, Spain, Sri Lanka, Sudan, Suriname, Sweden, Switzerland, Syrian Arab Republic, Tajikistan, Thailand, Timor-Leste, Togo, Tonga, Trinidad and Tobago, Tunisia, Turkey, Turkmenistan, Uganda, Ukraine, United Arab Emirates, United Kingdom of Great Britain and Northern Ireland, United Republic of Tanzania, United States of America, Uruguay, Uzbekistan, Vanuatu, Venezuela (Bolivarian Republic of), Viet Nam, Yemen, Zambia, Zimbabwe

**Countries within the lowest quintile of UNPD estimate of total population between 2000–2019 (42 countries)**

Andorra, Antigua and Barbuda, Bahamas, Bahrain (2000-2002), Barbados, Belize, Bhutan, Brunei Darussalam, Cabo Verde, Comoros, Cook Islands, Djibouti (2003-2019), Dominica, Equatorial Guinea (2000-2007), Fiji (2005, 2008-2019), Grenada, Guyana (2002-2019), Iceland, Kiribati, Luxembourg, Maldives, Malta, Marshall Islands, Micronesia (Federated States of), Monaco, Montenegro (2006-2019), Nauru, Niue, Palau, Qatar 2000-2004), Saint Kitts and Nevis, Saint Lucia, Saint Vincent and the Grenadines, Samoa, San Marino, Sao Tome and Principe, Seychelles, Solomon Islands, Suriname, Tonga, Tuvalu, Vanuatu

**Countries within the second quintile of UNPD estimate of total population between 2000–2019 (46 countries)**

Albania, Armenia, Bahrain (2003-2019), Bosnia and Herzegovina, Botswana, Central African Republic, Congo (2000-2018), Costa Rica (2000-2006, 2012-2019), Croatia (2007-2019), Cyprus, Djibouti (2000-2002), Equatorial Guinea (2008-2019), Eritrea, Estonia, Eswatini, Fiji (2000-2004, 2006-2007), Gabon, Gambia, Georgia (2005-2019), Guinea-Bissau, Guyana (2000, 2001), Ireland (2000-2010, 2012-2019), Jamaica, Kuwait, Latvia, Lebanon (2000, 2001), Lesotho, Liberia, Lithuania, Mauritania, Mauritius, Mongolia, Namibia, New Zealand, North Macedonia, Norway (2019), Oman, Panama, Qatar (2005-2019), Republic of Moldova (2002-2019), Singapore (2000-2005), Slovenia, Timor-Leste (2002-2019), Trinidad and Tobago, United Arab Emirates (2000-2004), Uruguay

**Countries within the third quintile of UNPD estimate of total population between 2000–2019 (51 countries)**

Austria, Azerbaijan, Belarus (2001-2019), Benin (2000-2017), Bolivia (Plurinational State of), Bulgaria, Burundi, Chad (2000-2005), Congo (2019), Costa Rica (2007-2011), Croatia (2000-2006), Cuba (2018, 2019), Czechia (2006-2019), Denmark, Dominican Republic, El Salvador, Finland, Georgia (2000-2004), Greece (2013-2019), Guinea (2000-2011), Haiti, Honduras, Hungary (2004-2019), Ireland (2011), Israel, Jordan, Kyrgyzstan, Lao People's Democratic Republic, Lebanon (2002-2019), Libya, Nicaragua, Norway (2000-2018), Papua New Guinea, Paraguay, Portugal (2010-2019), Republic of Moldova (2000, 2001), Rwanda (2000-2012), Senegal (2000), Serbia, Sierra Leone, Singapore (2006-2019), Slovakia, Somalia (2000-2003), South Sudan (2011-2019), Sweden, Switzerland, Tajikistan, Togo, Tunisia (2000-2009), Turkmenistan, United Arab Emirates (2005-2019)

**Countries within the fourth quintile of UNPD estimate of total population between 2000–2019 (48 countries)**

Afghanistan (2000-2010, 2012-2015), Angola, Australia, Belarus (2000), Belgium, Benin (2018, 2019), Burkina Faso, Côte d'Ivoire, Cambodia, Cameroon, Chad (2006-2019), Chile, Cuba (2000-2017), Czechia (2000-2005), Democratic People's Republic of Korea, Ecuador, Ghana, Greece (2000-2012), Guatemala, Guinea (2012-2019), Hungary (2000-2003), Iraq (2000-2008), Kazakhstan, Madagascar, Malawi, Malaysia, Mali, Morocco (2016-2019), Mozambique, Nepal, Netherlands, Niger, Peru (2009-2019), Portugal (2000-2009), Romania, Rwanda (2013-2019), Saudi Arabia, Senegal (2001-2019), Somalia (2004-2019), Sri Lanka, Syrian Arab Republic, Tunisia (2010-2019), Uganda (2000-2002), Uzbekistan (2003-2019), Venezuela (Bolivarian Republic of), Yemen, Zambia, Zimbabwe

**Countries within the highest quintile of UNPD estimate of total population between 2000–2019 (42 countries)**

Afghanistan (2011, 2016-2019), Algeria, Argentina, Bangladesh, Brazil, Canada, China, Colombia, Democratic Republic of the Congo, Egypt, Ethiopia, France, Germany, India, Indonesia, Iran (Islamic Republic of), Iraq (2009-2019), Italy, Japan, Kenya, Mexico, Morocco (2000-2015), Myanmar, Nigeria, Pakistan, Peru (2000-2008), Philippines, Poland, Republic of Korea, Russian Federation, South Africa, Spain, Sudan, Thailand, Turkey, Uganda (2003-2019), Ukraine, United Kingdom of Great Britain and Northern Ireland, United Republic of Tanzania, United States of America, Uzbekistan (2000-2002), Viet Nam

## Immunization coverage level

Countries were classified into four groups according to vaccination coverage level (below 80%; 80% to under 90%; 90% to under 95%; 95% and above) using average WHO/UNICEF Estimates on National Immunization Coverage (WUENIC) for DTP1 and DTP3 between 2017 and 2019, as of July 2020 [8]. The coverage ranges were chosen similar to the classification of district coverage levels in the WHO/UNICEF Joint Reporting Form [9].

**Countries with an average DTP1 and DTP3 WUENIC estimate between 2017–2019 below 80% (28 countries)**

Afghanistan, Angola, Cameroon, Central African Republic, Chad, Congo, Democratic Republic of the Congo, Equatorial Guinea, Ethiopia, Gabon, Guinea, Haiti, Lao People's Democratic Republic, Madagascar, Mali, Nigeria, Pakistan, Papua New Guinea, Philippines, Samoa, Somalia, South Africa, South Sudan, Suriname, Syrian Arab Republic, Ukraine, Venezuela (Bolivarian Republic of), Yemen

**Countries with an average DTP1 and DTP3 WUENIC estimate between 2017–2019 between 80% to under 90% (31 countries)**

Argentina, Austria, Benin, Bolivia (Plurinational State of), Bosnia and Herzegovina, Brazil, Djibouti, Ecuador, El Salvador, Guatemala, Guinea-Bissau, Indonesia, Iraq, Lebanon, Lesotho, Liberia, Libya, Marshall Islands, Mauritania, Mexico, Micronesia (Federated States of), Niger, Paraguay, Peru, Romania, San Marino, Solomon Islands, Timor-Leste, Togo, Vanuatu, Viet Nam

**Countries with an average DTP1 and DTP3 WUENIC estimate between 2017–2019 between 90% to under 95% (40 countries)**

Algeria, Armenia, Bahamas, Barbados, Belize, Bulgaria, Burkina Faso, Burundi, Cambodia, Canada, Colombia, Comoros, Côte d'Ivoire, Dominican Republic, Estonia, Eswatini, Finland, Gambia, Honduras, Iceland, India, Kenya, Lithuania, Malawi, Montenegro, Mozambique, Myanmar, Namibia, Nepal, New Zealand, North Macedonia, Panama, Republic of Moldova, Saint Lucia, Senegal, Sierra Leone, Tunisia, United Republic of Tanzania, Zambia, Zimbabwe

**Countries with an average DTP1 and DTP3 WUENIC coverage between 2017–2019 of 95% and above (95 countries)**

Albania, Andorra, Antigua and Barbuda, Australia, Azerbaijan, Bahrain, Bangladesh, Belarus, Belgium, Bhutan, Botswana, Brunei Darussalam, Cabo Verde, Chile, China, Cook Islands, Costa Rica, Croatia, Cuba, Cyprus, Czechia, Democratic People's Republic of Korea, Denmark, Dominica, Egypt, Eritrea, Fiji, France, Georgia, Germany, Ghana, Greece, Grenada, Guyana, Hungary, Iran (Islamic Republic of), Ireland, Israel, Italy, Jamaica, Japan, Jordan, Kazakhstan, Kiribati, Kuwait, Kyrgyzstan, Latvia, Luxembourg, Malaysia, Maldives, Malta, Mauritius, Monaco, Mongolia, Morocco, Nauru, Netherlands, Nicaragua, Niue, Norway, Oman, Palau, Poland, Portugal, Qatar, Republic of Korea, Russian Federation, Rwanda, Saint Kitts and Nevis, Saint Vincent and the Grenadines, Sao Tome and Principe, Saudi Arabia, Serbia, Seychelles, Singapore, Slovakia, Slovenia, Spain, Sri Lanka, Sudan, Sweden, Switzerland, Tajikistan, Thailand, Tonga, Trinidad and Tobago, Turkey, Turkmenistan, Tuvalu, Uganda, United Arab Emirates, United Kingdom of Great Britain and Northern Ireland, United States of America, Uruguay, Uzbekistan

## Birth registration level

Countries were classified into four groups according to birth registration level (below 80%; 80% to under 90%; 90% to under 95%; 95% and above). Estimates of birth registration levels, that is the percentage of children under age five whose births are registered, for 172 WHO Member States were retrieved from UNICEF, as of July 2020 [10]. The coverage ranges were chosen analogously to the classification of immunization coverage levels (see previous section). In case multiple estimates were available for the same country, only the latest estimate was considered.

**Countries with latest available UNICEF estimate of birth registration level below 80% (43 countries)**

Afghanistan, Angola, Bangladesh, Burkina Faso, Cambodia, Cameroon, Central African Republic, Chad, Côte d'Ivoire, Democratic Republic of the Congo, Equatorial Guinea, Eswatini, Ethiopia, Gambia, Ghana, Guinea, Guinea-Bissau, Indonesia, Kenya, Lao People's Democratic Republic, Lesotho, Liberia, Malawi, Mauritania, Mozambique, Namibia, Nepal, Niger, Nigeria, Pakistan, Papua New Guinea, Samoa, Senegal, Somalia, Sudan, Timor-Leste, Tuvalu, Uganda, United Republic of Tanzania, Vanuatu, Yemen, Zambia, Zimbabwe

**Countries with latest available UNICEF estimate of birth registration level between 80% to under 90% (17 countries)**

Benin, Botswana, Burundi, Comoros, Dominican Republic, Gabon, Haiti, India, Madagascar, Mali, Myanmar, Nicaragua, Paraguay, Rwanda, Sierra Leone, Solomon Islands, Togo

**Countries with latest available UNICEF estimate of birth registration level between 90% to under 95% (13 countries)**

Azerbaijan, Bolivia (Plurinational State of), Cabo Verde, Djibouti, Ecuador, Guyana, Honduras, Kiribati, Philippines, Saint Lucia, South Africa, Tonga, Venezuela (Bolivarian Republic of)

**Countries with latest available UNICEF estimate of birth registration level of 95% and above (99 countries)**

Albania, Algeria, Andorra, Argentina, Armenia, Australia, Austria, Bahrain, Barbados, Belarus, Belgium, Belize, Bhutan, Bosnia and Herzegovina, Brazil, Bulgaria, Canada, Chile, Colombia, Congo, Costa Rica, Croatia, Cuba, Cyprus, Czechia, Democratic People's Republic of Korea, Denmark, Egypt, El Salvador, Estonia, Finland, France, Georgia, Germany, Greece, Guatemala, Hungary, Iceland, Iran (Islamic Republic of), Iraq, Ireland, Israel, Italy, Jamaica, Japan, Jordan, Kazakhstan, Kyrgyzstan, Latvia, Lebanon, Lithuania, Luxembourg, Maldives, Malta, Marshall Islands, Mexico, Monaco, Mongolia, Montenegro, Morocco, Nauru, Netherlands, New Zealand, North Macedonia, Norway, Oman, Panama, Peru, Poland, Portugal, Qatar, Republic of Moldova, Romania, Russian Federation, San Marino, Sao Tome and Principe, Serbia, Singapore, Slovakia, Slovenia, Spain, Sri Lanka, Suriname, Sweden, Switzerland, Syrian Arab Republic, Tajikistan, Thailand, Trinidad and Tobago, Tunisia, Turkey, Turkmenistan, Ukraine, United Arab Emirates, United Kingdom of Great Britain and Northern Ireland, United States of America, Uruguay, Uzbekistan, Viet Nam

**Countries without UNICEF estimate of birth registration level (22 countries)**

Antigua and Barbuda, Bahamas, Brunei Darussalam, China, Cook Islands, Dominica, Eritrea, Fiji, Grenada, Kuwait, Libya, Malaysia, Mauritius, Micronesia (Federated States of), Niue, Palau, Republic of Korea, Saint Kitts and Nevis, Saint Vincent and the Grenadines, Saudi Arabia, Seychelles, South Sudan

## Fragile- and conflict-affected situations (FCS)

Since 2004, the World Bank groups its Member States according to the presence of institutional and social fragility and violent conflict. Information on FCS status (yes/no) for 192 WHO Member States was retrieved from the World Bank, as of July 2020 [6].

**Countries classified by the World Bank as fragile- and conflict-affected situations between 2004–2019 (55 countries)**

Afghanistan, Angola (2004-2011), Bosnia and Herzegovina (2008-2014), Burkina Faso (2018-2019), Burundi, Côte d'Ivoire (2004-2017), Cambodia (2004-2007), Cameroon (2007-2008, 2018-2019), Central African Republic, Chad, Comoros, Congo (2004-2012, 2016-2019), Democratic Republic of the Congo, Djibouti (2004-2008, 2015-2017), Eritrea, Gambia (2004-2008, 2014-2019), Georgia (2008-2010), Guinea (2004-2011), Guinea-Bissau, Haiti, Iraq (2009-2019), Kiribati (2006-2019), Lao People's Democratic Republic (2004-2007, 2019), Lebanon (2014-2019), Liberia, Libya (2011-2019), Madagascar (2012-2015), Malawi (2012), Mali (2012-2019), Marshall Islands (2010-2019), Mauritania (2005), Micronesia (Federated States of; 2010-2019), Mozambique (2016-2017, 2019), Myanmar, Nepal (2008-2012), Niger (2018-2019), Nigeria (2004-2005, 2018-2019), Papua New Guinea (2004-2006, 2008, 2015-2019), Sao Tome and Principe (2004-2009), Sierra Leone (2004-2016), Solomon Islands, Somalia, South Sudan (2011-2019), Sudan, Syrian Arab Republic (2011-2019), Tajikistan (2004, 2007-2009), Timor-Leste (2004-2014, 2017-2019), Togo (2004-2017), Tonga (2004-2008), Tuvalu (2011-2019), Uzbekistan (2004-2007), Vanuatu (2004-2006), Venezuela (Bolivarian Republic of; 2018-2019), Yemen (2007-2019), Zimbabwe

**Countries not classified by the World Bank as fragile- and conflict-affected situations between 2004–2019 (139 countries)**

Albania, Algeria, Andorra, Antigua and Barbuda, Argentina, Armenia, Australia, Austria, Azerbaijan, Bahamas, Bahrain, Bangladesh, Barbados, Belarus, Belgium, Belize, Benin, Bhutan, Bolivia (Plurinational State of), Botswana, Brazil, Brunei Darussalam, Bulgaria, Cabo Verde, Canada, Chile, China, Colombia, Cook Islands, Costa Rica, Croatia, Cuba, Cyprus, Czechia, Democratic People's Republic of Korea, Denmark, Dominica, Dominican Republic, Ecuador, Egypt, El Salvador, Equatorial Guinea, Estonia, Eswatini, Ethiopia, Fiji, Finland, France, Gabon, Germany, Ghana, Greece, Grenada, Guatemala, Guyana, Honduras, Hungary, Iceland, India, Indonesia, Iran (Islamic Republic of), Ireland, Israel, Italy, Jamaica, Japan, Jordan, Kazakhstan, Kenya, Kuwait, Kyrgyzstan, Latvia, Lesotho, Lithuania, Luxembourg, Malaysia, Maldives, Malta, Mauritius, Mexico, Monaco, Mongolia, Montenegro, Morocco, Namibia, Nauru, Netherlands, New Zealand, Nicaragua, Niue, North Macedonia, Norway, Oman, Pakistan, Palau, Panama, Paraguay, Peru, Philippines, Poland, Portugal, Qatar, Republic of Korea, Republic of Moldova, Romania, Russian Federation, Rwanda, Saint Kitts and Nevis, Saint Lucia, Saint Vincent and the Grenadines, Samoa, San Marino, Saudi Arabia, Senegal, Serbia, Seychelles, Singapore, Slovakia, Slovenia, South Africa, Spain, Sri Lanka, Suriname, Sweden, Switzerland, Thailand, Trinidad and Tobago, Tunisia, Turkey, Turkmenistan, Uganda, Ukraine, United Arab Emirates, United Kingdom of Great Britain and Northern Ireland, United Republic of Tanzania, United States of America, Uruguay, Viet Nam, Zambia

## Support by Gavi, the Vaccine Alliance

Countries were classified by whether they had received financial support from Gavi, the Vaccine Alliance. Information on funding (yes/no) in any year between 2001 and 2019 for all WHO Member States was retrieved from Gavi, the Vaccine Alliance, as of July 2020 [11].

**Countries that have received support by Gavi, the Vaccine Alliance, in any year between 2000–2019 (77 countries)**

Afghanistan, Albania, Angola, Armenia, Azerbaijan, Bangladesh, Benin, Bhutan, Bosnia and Herzegovina, Burkina Faso, Burundi, Cambodia, Cameroon, Central African Republic, Chad, China, Comoros, Congo, Côte d'Ivoire, Cuba, Democratic People's Republic of Korea, Democratic Republic of the Congo, Djibouti, Eritrea, Ethiopia, Gambia, Georgia, Ghana, Guinea, Guinea-Bissau, Guyana, Haiti, Honduras, India, Indonesia, Kenya, Kiribati, Kyrgyzstan, Lao People's Democratic Republic, Lesotho, Liberia, Madagascar, Malawi, Mali, Mauritania, Mongolia, Mozambique, Myanmar, Nepal, Nicaragua, Niger, Nigeria, Pakistan, Papua New Guinea, Plurinational State of Bolivia, Republic of Moldova, Rwanda, Sao Tome and Principe, Senegal, Sierra Leone, Solomon Islands, Somalia, South Sudan, Sri Lanka, Sudan, Tajikistan, Timor-Leste, Togo, Turkmenistan, Uganda, Ukraine, United Republic of Tanzania, Uzbekistan, Viet Nam, Yemen, Zambia, Zimbabwe

**Countries that have not received support by Gavi, the Vaccine Alliance, in any year between 2000–2019 (117 countries)**

Algeria, Andorra, Antigua and Barbuda, Argentina, Australia, Austria, Bahamas, Bahrain, Barbados, Belarus, Belgium, Belize, Botswana, Brazil, Brunei Darussalam, Bulgaria, Cabo Verde, Canada, Chile, Colombia, Cook Islands, Costa Rica, Croatia, Cyprus, Czechia, Denmark, Dominica, Dominican Republic, Ecuador, Egypt, El Salvador, Equatorial Guinea, Estonia, Eswatini, Fiji, Finland, France, Gabon, Germany, Greece, Grenada, Guatemala, Hungary, Iceland, Iran (Islamic Republic of), Iraq, Ireland, Israel, Italy, Jamaica, Japan, Jordan, Kazakhstan, Kuwait, Latvia, Lebanon, Libya, Lithuania, Luxembourg, Malaysia, Maldives, Malta, Marshall Islands, Mauritius, Mexico, Micronesia (Federated States of), Monaco, Montenegro, Morocco, Namibia, Nauru, Netherlands, New Zealand, Niue, North Macedonia, Norway, Oman, Palau, Panama, Paraguay, Peru, Philippines, Poland, Portugal, Qatar, Republic of Korea, Romania, Russian Federation, Saint Kitts and Nevis, Saint Lucia, Saint Vincent and the Grenadines, Samoa, San Marino, Saudi Arabia, Serbia, Seychelles, Singapore, Slovakia, Slovenia, South Africa, Spain, Suriname, Sweden, Switzerland, Syrian Arab Republic, Thailand, Tonga, Trinidad and Tobago, Tunisia, Turkey, Tuvalu, United Arab Emirates, United Kingdom of Great Britain and Northern Ireland, United States of America, Uruguay, Vanuatu, Venezuela (Bolivarian Republic of)

## Immunization reporting system

Information on national immunization reporting systems was obtained from WHO, as of July 2020 [8].

**Countries with a centralized immunization reporting system (180 countries)**

Afghanistan, Albania, Algeria, Angola, Antigua and Barbuda, Argentina, Armenia, Australia, Azerbaijan, Bahamas, Bahrain, Bangladesh, Barbados, Belarus, Belize, Benin, Bhutan, Bolivia (Plurinational State of), Bosnia and Herzegovina, Botswana, Brazil, Brunei Darussalam, Bulgaria, Burkina Faso, Burundi, Cabo Verde, Cambodia, Cameroon, Central African Republic, Chad, Chile, China, Colombia, Comoros, Congo, Cook Islands, Costa Rica, Côte d'Ivoire, Croatia, Cuba, Cyprus, Czechia, Democratic People's Republic of Korea, Democratic Republic of the Congo, Denmark, Djibouti, Dominica, Dominican Republic, Ecuador, Egypt, El Salvador, Equatorial Guinea, Eritrea, Estonia, Eswatini, Ethiopia, Fiji, Gabon, Gambia, Georgia, Ghana, Grenada, Guatemala, Guinea, Guinea-Bissau, Guyana, Haiti, Honduras, Hungary, Iceland, India, Indonesia, Iran (Islamic Republic of), Iraq, Ireland, Israel, Italy, Jamaica, Japan, Jordan, Kazakhstan, Kenya, Kiribati, Kuwait, Kyrgyzstan, Lao People's Democratic Republic, Latvia, Lebanon, Lesotho, Liberia, Libya, Lithuania, Madagascar, Malawi, Malaysia, Maldives, Mali, Malta, Marshall Islands, Mauritania, Mauritius, Mexico, Micronesia (Federated States of), Mongolia, Montenegro, Morocco, Mozambique, Myanmar, Namibia, Nauru, Nepal, Netherlands, New Zealand, Nicaragua, Niger, Nigeria, Niue, North Macedonia, Oman, Pakistan, Palau, Panama, Papua New Guinea, Paraguay, Peru, Philippines, Poland, Portugal, Qatar, Republic of Korea, Republic of Moldova, Romania, Russian Federation, Rwanda, Saint Kitts and Nevis, Saint Lucia, Saint Vincent and the Grenadines, Samoa, San Marino, Sao Tome and Principe, Saudi Arabia, Senegal, Serbia, Seychelles, Sierra Leone, Singapore, Slovakia, Slovenia, Solomon Islands, Somalia, South Africa, South Sudan, Spain, Sri Lanka, Sudan, Suriname, Syrian Arab Republic, Tajikistan, Thailand, Timor-Leste, Togo, Tonga, Trinidad and Tobago, Tunisia, Turkey, Turkmenistan, Tuvalu, Uganda, Ukraine, United Arab Emirates, United Kingdom of Great Britain and Northern Ireland, United Republic of Tanzania, Uruguay, Uzbekistan, Vanuatu, Venezuela (Bolivarian Republic of), Viet Nam, Yemen, Zambia, Zimbabwe

**Countries without a centralized immunization reporting system (14 countries)**

Andorra, Austria, Belgium, Canada, Finland, France, Germany, Greece, Luxemburg, Monaco, Norway, Sweden, Switzerland, and the United States of America

## Immunization schedule

Information on national immunization schedules was obtained from WHO, as of July 2020 [8].

**Countries with BCG routine birth dose in the national immunization schedule in all years with WHO membership between 1999–2019 (152 countries)**

Afghanistan, Albania, Algeria, Angola, Argentina, Armenia, Azerbaijan, Bangladesh, Belarus, Belize, Benin, Bhutan, Bolivia (Plurinational State of), Bosnia and Herzegovina, Botswana, Brazil, Brunei Darussalam, Bulgaria, Burkina Faso, Burundi, Cabo Verde, Cambodia, Cameroon, Central African Republic, Chad, Chile, China, Colombia, Comoros, Congo, Cook Islands, Costa Rica, Côte d'Ivoire, Croatia, Cuba, Democratic People's Republic of Korea, Democratic Republic of the Congo, Djibouti, Dominica, Dominican Republic, Ecuador, Egypt, El Salvador, Equatorial Guinea, Eritrea, Estonia, Eswatini, Ethiopia, Fiji, Gabon, Gambia, Georgia, Ghana, Guatemala, Guinea, Guinea-Bissau, Guyana, Haiti, Honduras, Hungary, India, Indonesia, Iran (Islamic Republic of), Iraq, Jamaica, Kazakhstan, Kenya, Kiribati, Kyrgyzstan, Lao People's Democratic Republic, Latvia, Lesotho, Liberia, Libya, Lithuania, Madagascar, Malawi, Malaysia, Maldives, Mali, Marshall Islands, Mauritania, Mauritius, Mexico, Micronesia (Federated States of), Mongolia, Montenegro, Morocco, Mozambique, Myanmar, Namibia, Nauru, Nepal, Nicaragua, Niger, Nigeria, Niue, North Macedonia, Oman, Pakistan, Panama, Papua New Guinea, Paraguay, Peru, Philippines, Poland, Qatar, Republic of Korea, Republic of Moldova, Romania, Russian Federation, Rwanda, Saint Kitts and Nevis, Saint Lucia, Saint Vincent and the Grenadines, Samoa, Sao Tome and Principe, Saudi Arabia, Senegal, Serbia, Seychelles, Sierra Leone, Singapore, Solomon Islands, Somalia, South Africa, South Sudan, Sri Lanka, Sudan, Sweden, Syrian Arab Republic, Tajikistan, Thailand, Timor-Leste, Togo, Tonga, Tunisia, Turkey, Turkmenistan, Tuvalu, Uganda, Ukraine, United Arab Emirates, United Republic of Tanzania, Uruguay, Uzbekistan, Vanuatu, Venezuela (Bolivarian Republic of), Viet Nam, Yemen, Zambia, Zimbabwe

**Countries without BCG routine birth dose in the national immunization schedule in all years with WHO membership between 1999–2019 (42 countries)**

Andorra, Antigua and Barbuda, Australia, Austria, Bahamas, Bahrain, Barbados, Belgium, Canada, Cyprus, Czechia, Denmark, Finland, France, Germany, Greece, Grenada, Iceland, Ireland, Israel, Italy, Japan, Jordan, Kuwait, Lebanon, Luxembourg, Malta, Monaco, Netherlands, New Zealand, Norway, Palau, Portugal, San Marino, Slovakia, Slovenia, Spain, Suriname, Switzerland, Trinidad and Tobago, United Kingdom of Great Britain and Northern Ireland, United States of America

# References

1. R Core Team. R: A Language and Environment for Statistical Computing. In: R Foundation for Statistical Computing [Internet]. 2020. Available: https://www.r-project.org

2. Brooks ME, Kristensen K, van Benthem KJ, Magnusson A, Berg CW, Nielsen A, et al. {glmmTMB} Balances Speed and Flexibility Among Packages for Zero-inflated Generalized Linear Mixed Modeling. R J. 2017;9: 378–400.

3. Lenth R. emmeans: Estimated Marginal Means, aka Least-Squares Means. 2020 [cited 24 Apr 2021]. Available: https://cran.r-project.org/package=emmeans

4. Lüdecke D. ggeffects: Tidy Data Frames of Marginal Effects from Regression Models. J Open Source Softw. 2018;3: 772. doi:10.21105/joss.00772

5. World Health Organization. List of WHO Member States. 2020 [cited 15 Jul 2020]. Available: https://www.who.int/countries

6. The World Bank. Classifying countries by income. 2021 fiscal year. In: World Development Indicators [Internet]. 2020 [cited 17 Aug 2020]. Available: https://datahelpdesk.worldbank.org/knowledgebase/articles/906519-world-bank-country-and-lending-groups

7. United Nations: Department of Economic and Social Affairs - Population Division. World Population Prospects 2019, Online Edition. New York; 2019 [cited 23 Feb 2021]. Available: https://population.un.org/wpp/Download/Standard/Population/

8. World Health Organization. Immunization database. Expanded Programme on Immunization (EPI). Department of Immunization, Vaccines and Biologicals (IVB). 2020 [cited 15 Jul 2020]. Available: https://immunizationdata.who.int

9. World Health Organization. WHO/UNICEF Joint Reporting Form on Immunization (JRF) - Online example. 2020 [cited 15 Jul 2020]. Available: https://www.who.int/immunization/monitoring_surveillance/routine/reporting/WHO_UNICEF_JRF_EN.xls

10. United Nations Children’s Fund. Birth Registration for Every Child by 2030: Are we on track? New York; 2019.

11. Gavi Alliance. Disbursements and commitments. 2020 [cited 30 Sep 2020]. Available: https://www.gavi.org/programmes-impact/our-impact/disbursements-and-commitments
